# Long-Term Trajectories in Weight and Health Outcomes Following Multidisciplinary Publicly Funded Bariatric Surgery in Patients with Clinically Severe Obesity (≥ 3 Associated Comorbidities): A Nine-Year Prospective Cohort Study in Australia

**DOI:** 10.3390/jcm11154466

**Published:** 2022-07-31

**Authors:** Michelle M.C. Tan, Xingzhong Jin, Craig Taylor, Adrian K. Low, Philip Le Page, David Martin, Ang Li, David Joseph, Nic Kormas

**Affiliations:** 1 Boden Initiative, Central Clinical School, Sydney School of Medicine, Charles Perkins Centre, Faculty of Medicine and Health, The University of Sydney, Camperdown, NSW 2006, Australia; ang.li5@unimelb.edu.au; 2 Department of Endocrinology & Metabolism, Concord Repatriation General Hospital, Sydney Local Health District, Concord, NSW 2139, Australia; 3 Diabetes, Metabolism & Obesity Translational Research Unit, Camden Hospital, South Western Sydney Local Health District, Camden, NSW 2570, Australia; 4 Department of Endocrinology, Royal Prince Alfred Hospital, Sydney Local Health District, Camperdown, NSW 2050, Australia; 5 Centre for Big Data Research in Health, Faculty of Medicine, University of New South Wales, Kensington, NSW 2052, Australia; xingzhong.jin@unsw.edu.au; 6 Institute of Bone and Joint Research, Kolling Institute, The University of Sydney, St Leonards, NSW 2064, Australia; 7 Upper GI Surgery Department, Concord Repatriation General Hospital, Sydney Local Health District, Concord, NSW 2139, Australia; drtaylor@oclinic.com.au (C.T.); phillepage11@gmail.com (P.L.P.); davidmartin72@hotmail.com (D.M.); davejoseph1@yahoo.com.au (D.J.); 8 Department of Orthopaedic Surgery, Faculty of Medicine and Health, The University of Sydney, Sydney Adventist Hospital, Wahroonga, NSW 2076, Australia; adrianklow@gmail.com; 9 Hepatobiliary & Upper GIT Surgery Department, Royal Prince Alfred Hospital, Sydney Local Health District, Camperdown, NSW 2050, Australia; 10 NHMRC Centre of Research Excellence in Healthy Housing, Centre for Health Policy, Melbourne School of Population and Global Health, The University of Melbourne, Parkville, VIC 3010, Australia

**Keywords:** bariatric surgery, long-term outcomes, publicly funded, multidisciplinary management, clinically severe obesity

## Abstract

Background: Real-world data on long-term (> 5 years) weight loss and obesity-related complications after newer bariatric surgical procedures are currently limited. The aim of this longitudinal study was to examine the effectiveness and sustainability of bariatric surgery in a cohort with clinically severe obesity in a multidisciplinary publicly funded service in two teaching hospitals in New South Wales, Australia. Methods: Patients were adults with complex clinically severe obesity with a BMI ≥ 35 kg/m^2^ and at least three significant obesity-related comorbidities, who underwent bariatric surgeries between 2009 and 2017. Detailed obesity-related health outcomes were reported from annual clinical data and assessments for up to 9 years of follow-up. Data were also linked with the national joint replacement registry. Results: A total of 65 eligible patients were included (mean, 7; range, 3–12 significant obesity-related comorbidities); 53.8% female; age 54.2 ± 11.2 years, with baseline BMI 52.2 ± 12.5 kg/m^2^ and weight 149.2 ± 45.5 kg. Most underwent laparoscopic sleeve gastrectomy (80.0%), followed by laparoscopic adjustable gastric banding (10.8%) and one anastomosis gastric bypass (9.2%). Substantial weight loss was maintained over 9 years of follow-up (*p* < 0.001 versus baseline). Significant total weight loss (%TWL ± SE) was observed (13.2 ± 2.3%) following an initial 1-year preoperative intensive lifestyle intervention, and ranged from 26.5 ± 2.3% to 33.0 ± 2.0% between 1 and 8 years following surgery. Type 2 diabetes mellitus (T2DM), osteoarthritis-related joint pain and depression/severe anxiety were the most common metabolic, mechanical and mental health comorbidities, with a baseline prevalence of 81.5%, 75.4% and 55.4%, respectively. Clinically significant composite cumulative rates of remission and improvement occurred in T2DM (50.0–82.0%) and hypertension (73.7–82.9%) across 6 years. Dependence on continuous positive airway pressure treatment in patients with sleep-disordered breathing fell significantly from 63.1% to 41.2% in 6 years. Conclusion: Bariatric surgery using an intensive multidisciplinary approach led to significant long-term weight loss and improvement in obesity-related comorbidities among the population with clinically complex obesity. These findings have important implications in clinical care for the management of the highest severity of obesity and its medical consequences. Major challenges associated with successful outcomes of bariatric surgery in highly complex patients include improving mental health in the long run and reducing postoperative opioid use. Long-term follow-up with a higher volume of patients is needed in publicly funded bariatric surgery services to better monitor patient outcomes, enhance clinical data comparison between services, and improve multidisciplinary care delivery.

## 1. Introduction

Obesity is a staggering global epidemic with a disproportionate rise in class III obesity (defined by body mass index (BMI) of ≥ 40 kg/m^2^), causing a substantial burden on healthcare systems worldwide. There are approximately one million adults in Australia with severe levels of obesity, namely clinically severe obesity, which is defined as class III obesity alone or a BMI of ≥ 35 kg/m^2^ with major obesity-related comorbidities. In an effort to address this epidemic, several health departments of Australian states and territories have launched multidisciplinary specialist obesity services (i.e., specialist hospital-based multidisciplinary healthcare services for the management of obesity and its associated medical conditions), with a few successfully establishing publicly funded bariatric surgery services to manage socioeconomically disadvantaged and highly complex patients with clinically severe obesity [1,2,3]. However, the vast majority of bariatric procedures (93.9%) were performed in private hospitals, with critically low access in the resource-constrained public healthcare system [4]. As such, this unique cohort of patients is not well-researched.

In the past two decades, bariatric surgery has universally become an increasingly popular treatment for clinically severe obesity [5,6,7,8,9,10]. It has been generally accepted as the most effective treatment, with superior results in weight loss, mortality risk reduction, cardiovascular risk reduction and resolution or improvement of obesity-related comorbidities, contributing to enhanced quality of life and increased life expectancy [8,11,12,13,14]. However, most evaluations of bariatric surgical outcomes have been hampered by inadequate and incomplete long-term follow-up or significantly contrast procedures no longer performed today, such as the nonadjustable gastric band and vertical banded gastroplasty reported in the well-respected Swedish Obese Subjects (SOS) study [15,16]. The Longitudinal Assessment of Bariatric Surgery (LABS) Consortium from the United States has published 3- and 7-year outcomes from two bariatric surgical procedures, adjustable gastric banding (AGB) and Roux-en-Y gastric bypass (RYGB) [7,8]. However, these reports do not include the widely accepted, stand-alone bariatric operations that are gaining popularity and acceptance among bariatric surgeons: sleeve gastrectomy (SG) and one anastomosis gastric bypass (OAGB). There are continued gaps in evidence about the long-term effectiveness and sustainability (i.e., > 5 years) of these newer operations that have supplanted other procedures [3,17,18]. Besides temporally, there is also significant variability in outcome measures within different study populations; highly severe populations remain precluded and largely unexplained. Other endeavours have generally focused on one aspect of bariatric surgical outcomes, such as weight loss [19].

In recent years, bariatric surgery has also proven to offer metabolic and mechanical benefits, resulting in a shift from mere weight management as the primary focus of bariatric surgery to the improvement or resolution of obesity-related comorbidities. However, more information and an improved understanding of the longer-term sustainability of control of comorbidities associated with obesity after bariatric procedures are needed. It is important to understand whether remission and improvement in obesity-related comorbidities are sustainable over time, and whether there are worsening or emergent incident conditions after surgical treatments. Many previous studies have not reported progressive and combined measures (both medication use and blood tests) of obesity-related comorbidities over a long-term period [3,20,21]. A recent systematic review showed that the effects of bariatric surgery on mental health conditions are contradictory [22]. High rates of mental health conditions are relatively common in patients with extreme obesity—as high as 30–40% in bariatric surgery candidates [23,24]—yet long-term data, particularly beyond 3 years post-surgery, are also limited on mental illness after bariatric surgery [22]. The data on these health outcomes in highly complex populations undergoing bariatric procedures is needed in real-world clinical settings.

Reports on outcomes of publicly funded bariatric surgery are lacking yet crucial in determining its practicality, challenges and utility. Observing the changes in this broad range of important outcome measures at annual timepoints over a long period would advance the literature and improve the understanding of the impact of bariatric surgery on metabolic diseases, mechanical complications and mental health. Consequently, this would aid in defining evidence-based targets, inform decisions for publicly funded bariatric surgery services in Australia and worldwide, and facilitate optimal allocation of healthcare resources. In line with such rationales, the effectiveness and sustainability of bariatric surgery on type 2 diabetes mellitus (T2DM), hypertension, hyperlipidaemia, osteoarthritis (OA)-related joint pain, sleep-disordered breathing, depression and severe anxiety were critically examined and discussed in this study. We closely evaluated the effectiveness of newer bariatric surgical procedures on weight loss and these obesity-related comorbidities in a multidisciplinary publicly funded service in Australia in the setting of clinically complex obesity with at least three comorbidities over a long-term follow-up duration.

## 2. Methods

### 2.1. Study Design

This study involved a retrospective and prospective data collection of eligible patients with clinically severe obesity who underwent publicly funded bariatric surgery between December 2009 and December 2017. We carried out the present multidisciplinary, multistage and multisurgeon (C.T., D.M., P.L.P. and D.J.) bariatric surgery research in two public hospitals in Greater Sydney, New South Wales (NSW), Australia that are providing publicly funded care: Concord Repatriation General Hospital (CRGH) and Camden Hospital. All patients were operated at our own single local bariatric surgical facility, CRGH, which is also a major teaching hospital, after they had been assessed and prepared with a 1-year preoperative intensive physician-led lifestyle program. Adjunct treatments offered in this prehabilitation program included structured exercise prescription with on-site group exercise classes, exercise prescription for community-based activity, psychological therapies and medical consultations, and individual dietary interventions as needed. Patients then underwent a formal, comprehensive, multidisciplinary assessment to determine their fitness for bariatric surgery by an endocrinologist, bariatric surgeon, clinical nurse consultant, exercise physiologist/physiotherapist, dietitian and psychologist/psychiatrist. After bariatric surgery, they were followed-up annually and assessed for up to 8 years postoperatively.

### 2.2. Patients

The inclusion criteria for referral and enrolment in this publicly funded bariatric surgery service were an age of 18 years or older, and an initial BMI ≥ 35 kg/m^2^ with multiple significant obesity-related comorbidities (e.g., type 2 diabetes mellitus (T2DM), severe obstructive sleep apnoea (OSA)/obesity hypoventilation syndrome (OHS), osteoarthritis (OA) with functional impairment, and non-alcoholic fatty liver disease). Exclusion criteria were irreversible endocrine or other disorders that can cause obesity; current drug or alcohol abuse; uncontrolled, severe psychiatric illness; pregnancy; and inability to attend postsurgical follow-up appointments.

Research assessments were conducted by review and data extraction from electronic and paper-based medical records, interviews, questionnaires, telephone calls, and mailings. The available blood test results for biochemistry parameters were extracted from both electronic and paper-based hospital records (whichever were available). In the absence of laboratory results in hospital records, phone call attempts were made to request a copy of the patient’s blood test findings from the centralized pathology laboratories to be emailed and faxed to the clinics. The laboratory results were also accessed and obtained from the online NSW Health Pathology portal if any readings were available in the online portal but not other sources.

### 2.3. Outcome Measures

#### 2.3.1. Weight Change

Weight change, the primary endpoint of the study, was evaluated based on weight loss in percent of total weight loss (%TWL) and BMI loss, from the initial (i.e., entry to clinic) and preoperative baseline (in which baseline weight was measured closest to the time of bariatric surgery) to year 8 postoperation. The weight loss indices were calculated according to standard equations. Body mass index (BMI) was categorised according to the World Health Organization (WHO) classification [25]; all study patients had class III obesity upon initial consultation. Following the integrated preoperative prehabilitation at the clinics and immediately before undergoing bariatric surgery, patients were reassessed and classified based on their preoperative BMI.

#### 2.3.2. Definition and Postoperative Course of Obesity-Related Comorbidities

The predefined secondary endpoints—changes in the status of comorbidities strongly associated with obesity—were assessed at each follow-up visit over 6 years based on symptoms, laboratory findings, physical measures and medication use. In addition to prevalence rates, the postoperative courses of comorbidities were defined as: in remission (asymptomatic and medications no longer needed), improved (reduction in the number of active medications and/or fewer symptoms), persisting (same symptoms and equivalent medications as before bariatric surgery) or worsened (increase in therapy or a change from non-insulin treatment to insulin use in the case of T2DM) for T2DM, hypertension and hyperlipidaemia. The incidence of comorbidity was defined as patients without the comorbidity at preoperative baseline who newly developed the comorbidity after bariatric surgery. As this study was not intended to measure medication adherence but to reflect complexity of comorbidities, medication dosage was not factored into the calculation.

#### 2.3.3. Type 2 Diabetes Mellitus Status

Preoperative T2DM status was determined based on physician’s diagnosis, prescription of any diabetes medication (insulin, oral hypoglycaemic agents (OHAs) and/or injectable diabetes medication) or a plasma glycosylated haemoglobin (HbA_1c_) measure of ≥ 6.5%. If the HbA_1c_ level was unavailable, fasting blood glucose (FBG) of ≥ 7.0 mmol/L was used. T2DM remission status after bariatric surgery was modified from the American Diabetes Association criteria [26]. Remission of T2DM was determined as a HbA_1c_ < 6.5% and a FBG < 7.0 mmol/L in the absence of antidiabetic medications. Improvement in T2DM was defined as improved parameters in terms of reduced number of medications or a change from insulin use to non-insulin treatment. Persisting T2DM status was defined by unchanged antidiabetic medications calculated from preoperative baseline. Worsening T2DM was assessed as newly prescribed antidiabetic medications after the surgery, increased number of OHAs and/or changing from OHAs to insulin use. Patients presenting with polycystic ovarian syndrome who did not meet the laboratory criteria for T2DM and were not on an antidiabetic medication other than metformin were not considered to have T2DM. Incident T2DM was defined as any new-onset T2DM not present at preoperative baseline that developed throughout the 6-year postoperative period.

#### 2.3.4. Hypertension Status

Hypertension status was determined based on an abnormal increase in blood pressure (BP) with a systolic/diastolic BP ≥ 140/90 mmHg or treatment with antihypertensive drugs. Hypertension remission post-surgery was defined as normotensive (< 140/90 mmHg) without any antihypertensive therapy according to the American Society for Metabolic and Bariatric Surgery criteria [27]. Improvement in hypertension was defined as fewer antihypertensive therapies across timepoints, whereas persisting status was indicated by the same number of medications as before bariatric surgery. Worsened hypertension indicated an increased number of therapies in reference to presurgical status. Patients with a diagnosis of heart failure or atrial fibrillation who were treated with beta-blockers for their cardiac problems rather than for hypertension were excluded from analysis in this study.

#### 2.3.5. Hyperlipidaemia Status

Hyperlipidaemia status was determined based on a total cholesterol level ≥ 5.2 mmol/L (200 mg/dL), low-density lipoprotein cholesterol (LDL-C) ≥ 3.5 mmol/L (130 mg/dL), high-density lipoprotein cholesterol (HDL-C) ≤ 1.0 mmol/L (40 mg/dL), triglycerides ≥ 1.7 mmol/L (150 mg/dL) and/or treatment with lipid-lowering agents (i.e., statin, fibrate and ezetimibe). At the annual follow-ups, hyperlipidaemia remission required a complete return to normal lipid panel (of all the four serum lipid subfractions) with cessation of all lipid-lowering drugs [27]. Improvement in hyperlipidaemia was defined as a reduction in the number of prescribed lipid-lowering agents. Persisting hyperlipidaemia was defined as an unchanged number of lipid-lowering medications, and worsened status was indicated by a higher number of lipid-lowering agents.

#### 2.3.6. Sleep-Disordered Breathing Status

The status of preoperative sleep-disordered breathing, which consists of OSA and OHS, was determined based on a respiratory and sleep physician’s diagnosis according to previous diagnostic overnight inpatient polysomnography, as well as review of continuous positive airway pressure (CPAP) or bilevel positive airway pressure (BiPAP) device requirements. In view that the objective measurement, polysomnography, could not be performed for all patients, remission was not defined for sleep-disordered breathing. Instead, an improvement was defined as discontinuation of CPAP or BiPAP use, in addition to physicians’ diagnoses of decreased symptoms or normalized sleep patterns. This status adheres to both the objective and subjective improvement guidelines of the American Society for Metabolic and Bariatric Surgery [27]. Moreover, adherence to CPAP or BiPAP device use among the study patients was retrieved to eliminate self-discontinuation of the devices.

#### 2.3.7. Osteoarthritis-Related Joint Pain Status

Osteoarthritis (OA) was diagnosed either radiologically or clinically by a physician. Patients were evaluated for the presence of pain in weight-bearing joints (including hips, knees and lumbosacral spine) and whether any analgesic medications were required. In particular, opioid use was defined as a prescription of regular or daily opioid analgesics. Furthermore, data linkage with the Australian Orthopaedic Association National Joint Replacement Registry (AOANJRR) was conducted. Since its inception, the AOANJRR has collected data on almost 100% of primary and revision arthroplasty procedures performed in Australia.

#### 2.3.8. Depression/Severe Anxiety Status

Depression and severe anxiety were classified based on psychiatrists’ diagnoses, patient history/symptoms and/or psychologists’ assessments, as well as the use of pharmacotherapy (antidepressants, antianxiety agents or antipsychotic agents, as well as mood stabilisers) or ongoing non-pharmacological treatment for mental health illness, such as cognitive behavioural treatment. As depression and severe anxiety are often interrelated, we merged the two conditions into a single variable—namely, depression/severe anxiety—in the analysis.

### 2.4. Statistical Analyses

Descriptive statistics summarize baseline characteristics for the overall sample, each bariatric surgical procedure and by follow-up year. We provide an overview of 9-year descriptive weight change from initial clinic visit (i.e., 1 year prior to surgery, on lifestyle modifications, medical consultations and very low-energy diet (VLED)) to pre-surgery through 8 years following bariatric surgery. The mortality and national total joint arthroplasty (TJA) data from the AOANJRR were evaluated from the time of bariatric surgery until the 8th year of follow-up. Due to the small number of patients at years 7 and 8 post-surgery, the detailed comorbidity outcomes up to 6 years post-surgery were calculated, analysed and reported. The bootstrap 95% confidence intervals (CIs) for the comorbidity statuses in this study were computed by the bias-corrected and accelerated (BCa) method. Frequencies and percentages (%) were reported for categorical variables. Means and standard deviation (SD), as well as median and interquartile range (IQR), were reported for continuous data. Normality was assessed visually using the Shapiro–Wilk test. One-way ANOVA with post hoc Bonferroni test and paired-sample t-test were used for continuous variables. Chi-squared test and McNemar test were used for categorical variables.

The estimated marginal means of the BMI and %TWL modelled from linear mixed-effects models for indicator time after the lifestyle interventions and bariatric surgery were reported to account for reductions in sample size over time. We required that the smallest trajectory group include at least 5% of our total sample size (n = 3) at any follow-up timepoint; therefore, the statistical models were fitted up to 8 years post-surgery. The longitudinal analyses constructed from the linear mixed-effects models were based on the restricted maximum likelihood (REML) method, with a person-level random intercept (i.e., subject ID) included. The modelling included fixed effect terms for age at time of surgery, sex, race and clinic visits. A repeated measures term was included for the visits within each subject to take into account multiple observations over time, expressed as continuous data. Complete case analysis was conducted. The model fit was determined using Akaike’s information criterion (AIC). The modelled trajectory was plotted, with bars indicating the 95% CI of the modelled %TWL.

Generalised linear mixed models accounting for random effects at the patient level were fitted to model the longitudinal changes in metabolic health outcomes over time (i.e., T2DM, hypertension and hyperlipidaemia), expressed as categorical data comprising the four disease statuses (i.e., remission, improvement, persistence and worsening). Multinomial distribution with a generalised logit link function was applied, with multicategory responses of the four disease statuses of the entire cohort representing the target over a period of 6 years post-surgery. Fixed effect terms for age at time of surgery, sex, race and annual clinic visits were included. A random intercept for subject ID was included to account for multiple observations across years within each subject.

Data analyses were performed using IBM SPSS Statistics (Version 28, Armonk, NY, USA) and STATA/MP (Version 17, Stata Corp LP, College Station, TX, USA). All reported *p* values are 2-sided. A *p* value less than 0.05 was considered statistically significant.

## 3. Results

### 3.1. Study Cohort

Overall, a total of 65 patients suffering from clinically severe obesity were offered bariatric surgery between 2009 and 2017, with sleeve gastrectomy (SG) (80.0%, n = 52) as the most commonly performed index bariatric procedure, followed by laparoscopic adjustable gastric banding (LAGB) (10.8%) and one anastomosis gastric bypass (OAGB) (9.2%). All bariatric surgical operations were performed laparoscopically with no conversion to open. The average length of hospital stay was three days (range, 1–8 days). Mean follow-up time after bariatric surgery was four years. Extremely high rates of coexistence of comorbidities were observed; all patients had at least three significant obesity-related complications before surgery (mean, 7; range, 3–12).

At the initial medical review, the mean BMI (± SD) of the patients was 52.2 ± 12.5 kg/m^2^ (range, 35.0–116.7 kg/m^2^), corresponding to 149.2 ± 45.5 kg. Mean age at the time of surgery was 54 years (range, 21–72 years), with a mean preoperative baseline BMI decreased to 45.8 ± 8.8 kg/m^2^ (range, 33.6–64.0 kg/m^2^) (131.1 ± 34.0 kg) following the preoperative intensive lifestyle intervention. At baseline, the majority of patients were Caucasian; more than half of the patients were female and were current or ex-smokers, respectively. Socioeconomic disadvantage was prevalent among the study population, whereby 56.9% were unemployed and on government support payments.

Table 1 details the baseline characteristics of the study patients according to their primary procedures.

### 3.2. Weight Changes

Weight outcomes were charted from entry to clinics up to 8 years postoperative, accounting for preoperative baseline factors and the nature of the data. The observed BMI by follow-up timepoints from initial clinic visit to preoperative baseline through 8 years of follow-up after bariatric surgery are depicted in Table 2. Mean weight loss during the prehabilitation with lifestyle and very low-energy diet (VLED) interventions approximately one year before bariatric surgery was 18.1 kg, corresponding to 6.3 kg/m^2^. As shown in Table 2, there were further significant weight changes from preoperative baseline to each post-surgical visit during the observation period. The greatest actual weight loss occurred within the first 18 postoperative months (34.5 kg/m^2^, corresponding to 97.9 kg), followed by a slight regain, and remaining constant through the final follow-up observations. The weight recidivism from the weight loss nadir was observed between years 2 and 8 post-surgery. This observation at year 1.5 post-surgery is unique, as it has rarely been observed in previous studies, with only annual interval measurements reported.

**Figure 1:**
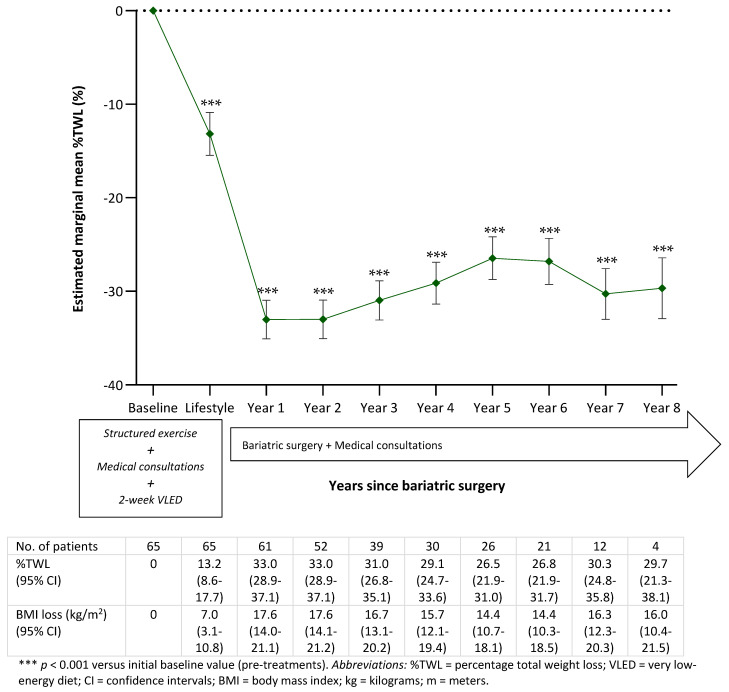
Modelled percentage total weight loss (%) from initial clinic visit (i.e., 1 year prior to surgery, with lifestyle modifications and medical consultations) to preoperation and year 8 post-bariatric surgery. *The overall estimated marginal mean weight loss at each visit over time modelled from the mixed-effects model with random effects, taking into account the repeated measures nature of the data. Lines indicate modelled weight change from baseline based on mixed models adjusted for baseline factors (age at time of surgery, sex and race). A negative value represents weight loss based on pre-surgery weight. Data markers (estimated marginal mean values) indicate weight change data. Error bars represent the 95% CI*.

Figure 1 represents the overall modelled weight change of the study patients by post-surgical follow-up year. This consists of the %TWL and BMI loss from initial clinic visits through baseline to 8 years after the operation. The linear mixed-effects model revealed that substantial and steady weight loss was achieved after the preoperative lifestyle interventions and bariatric surgery over 9 years of follow-up (*p* < 0.001 versus baseline), with a significant %TWL (± SE) of 13.2 ± 2.3% during initial lifestyle intervention and 33.0 ± 2.0% at 1 year, 33.0 ± 2.1% at 2 years, 31.0 ± 2.1% at 3 years, 29.1 ± 2.2% at 4 years, 26.5 ± 2.3% at 5 years, 26.8 ± 2.5% at 6 years, 30.3 ± 2.7% at 7 years and 29.7 ± 3.3% at 8 years following surgery. This corresponds to a change in BMI (± SE) of 7.0 ± 1.9 kg/m^2^ prior to bariatric surgery and 17.6 ± 1.8 kg/m^2^ at year 1, 17.6 ± 1.8 kg/m^2^ at year 2, 16.7 ± 1.8 kg/m^2^ at year 3, 15.7 ± 1.8 kg/m^2^ at year 4, 14.4 ± 1.9 kg/m^2^ at year 5, 14.4 ± 2.0 kg/m^2^ at year 6, 16.3 ± 2.0 kg/m^2^ at year 7 and 16.0 ± 2.4 kg/m^2^ at year 8 postoperatively.

Overall, following the lifestyle interventions and at the start of the surgical treatment, patients experienced rapid and maximum total weight loss in the first 2 years after bariatric surgery (Figure 1). From 2-year level, modelling began to demonstrate some modest weight regain, stabilizing at years 7 and 8. In summary, the study cohort significantly achieved and maintained a successful marginal estimated mean weight loss over each timepoint to their last observation (*p* < 0.001), with a net weight loss of 29.7 ± 3.3%TWL at postoperative year 8. That being said, the findings need to be interpreted cautiously in light of the reduced observations at 7 and 8 years following bariatric surgery.

### 3.3. Prevalence of Major Obesity-Related Comorbidities

**Figure 2:**
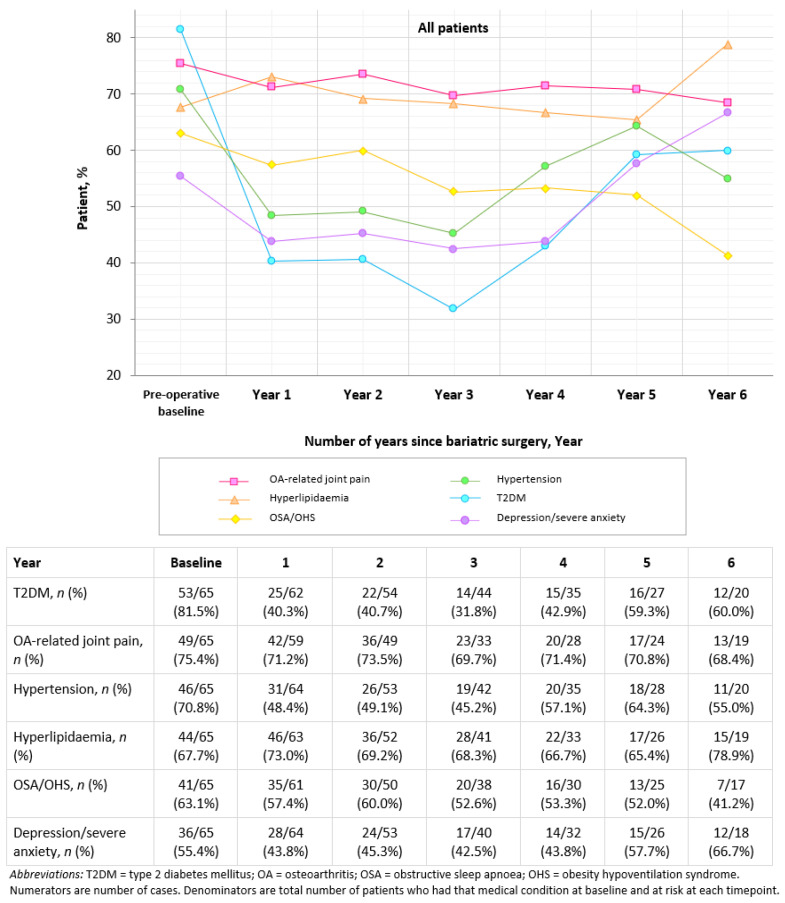
Prevalence of obesity-related comorbidities preoperatively and at 1–6 years following bariatric surgery (%).

Figure 2 shows the prevalence of obesity-related comorbidities of all patients at annual follow-up timepoints. At preoperative baseline, there were high rates of all obesity-related comorbidities. These include T2DM, OA-related joint pain, hypertension, hyperlipidaemia, OSA/OHS and depression/severe anxiety. The prevalence of OA-related joint pain and OSA/OHA declined consistently from baseline to the last timepoints. The other obesity-related comorbidities followed a quadratic trend, which declined dramatically from baseline to the first year post-surgery, followed by an increase in the comorbidity prevalence during years 2 through 6 to a state better than pre-surgery, except for depression/severe anxiety and hyperlipidaemia, which exceeded baseline levels as time progressed.

### 3.4. Description of Changes in Comorbidities with Weight Loss

The clinical profiles presented in Table 3 for the entire study population, regardless of baseline metabolic diseases, demonstrate that the postoperative diastolic BP measurements, glycaemic control (i.e., HbA_1c_), total cholesterol, triglyceride and HDL-C levels were improved relative to the baseline for the whole study population, whereas systolic BP and FBG levels were higher than those at the preoperative timepoint only at year 6 post-surgery.

### 3.5. Changes in Comorbidity Statuses

**Figure 3:**
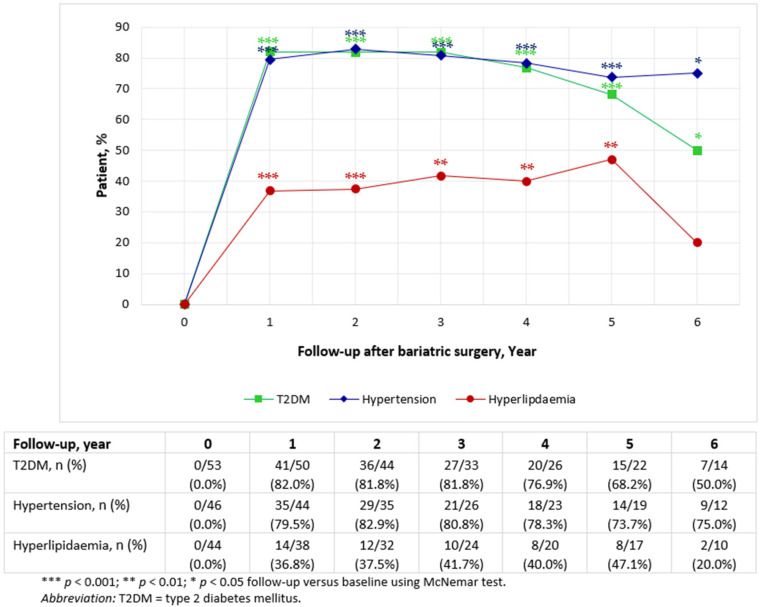
Yearly remission and improvement rates of T2DM, hypertension and hyperlipidaemia following bariatric surgery.

The progressive changes in statuses of metabolic diseases with weight loss were also generated based on clinical measures, biochemical tests, medication use and physicians’ examinations. The illustrations, proportions and 95% CIs of the postoperative statuses, blood tests, medication profiles and clinical measurements, are reported in Figure 3 and Table 4, Table 5 and Table 6.

Table 4, Table 5 and Table 6 display the annual changes in T2DM, hypertension and hyperlipidaemia statuses after bariatric surgery at each follow-up period, supplemented with detailed changes in clinical and biochemical characteristics.

#### 3.5.1. Type 2 Diabetes Mellitus

At baseline, 53 of 65 patients (81.5%) were diagnosed with T2DM (mean duration of diabetes, 9.7 ± 7.3 years); 20 of the patients with T2DM (37.7%) were receiving insulin treatment, whereas 45 (84.9%) were on glucose-lowering agents, with a mean elevated HbA_1c_ of 7.5% (Table 4). The resultant post-surgical weight loss following bariatric surgery substantially and rapidly resolved and improved T2DM, with 50.0–82.0% of patients experiencing significant remission and improvement of their T2DM during the 6-year follow-up period (Figure 3). The remission and improvement rates decreased over time but remained high in the long term. The composite cumulative remission and improvement rate was also calculated; 37 of 53 patients achieved a cumulative remission and improvement rate of T2DM following bariatric surgery to their last observations. This is equivalent to nearly three-quarters of those who reported T2DM at baseline (69.8%). In other words, although there appeared to be some declination in T2DM remission over time, most of the patients with T2DM who underwent bariatric surgery were still in remission and improvement at their last observations, whereas the worsening and incidence rates were extremely low. It is worth noting that there was no new onset of T2DM requiring antidiabetic medications during the long-term follow-up period. With respect to glycaemic control and medication breakdowns, a reduction in HbA_1c_ level from baseline was observed at all timepoints after bariatric surgery (Table 4). Mean FBG responded well between years 1 and 5 post-surgery but did not reduce substantially at year 6 compared to the preoperative baseline. Antidiabetic medication use significantly reduced post-surgery, with only less than one-fifth of patients requiring insulin at year-6 follow-up. Furthermore, a significant reduction in the number of oral drugs was observed over time.

#### 3.5.2. Hypertension

Changes in hypertension statuses are presented in Table 5. Among 70.8% of patients who underwent bariatric surgery and had hypertension at pre-surgical baseline, complete remission of hypertension was achieved in one-third over 6 years with no indication of hypertension following bariatric surgery, and improvement in another higher proportions of patients throughout the follow-up period. There appeared to be a similar but better trend to T2DM in hypertension remission and improvement rates; most patients maintained much of their remission and improvement in hypertension throughout the long-term follow-up period (73.7–82.9%) (Figure 3). Compared to the preoperative baseline, antihypertensive medications were discontinued in significant proportions of the patients, with a mean number of 2 preoperative antihypertensive drugs reduced to between 0.9 and 1.3 during the 6 years of postoperative follow-up (Table 5). The composite cumulative remission and improvement rate of hypertension to the final observations was as high as 74.0% (i.e., 34 of 46 patients with hypertension at baseline).

**Table 5:** Yearly remission, improved, persisting and worsened rates of hypertension following bariatric surgery ^b^.

Follow-Up, Year	0	1	2	3	4	5	6
**Remission**, n (%)		14/44 (31.8%)	11/35 (31.4%)	10/26 (38.5%)	7/23 (30.4%)	5/19 (26.3%)	3/12 (25.0%)
(95% CI)		(20.5–43.2)	(20.0–42.9)	(23.1–53.8)	(17.4–47.8)	(10.5–42.1)	(8.3–41.7)
**Improved**, n (%)		21/44 (47.7%)	18/35 (51.4%)	11/26 (42.3%)	11/23 (47.8%)	9/19 (47.4%)	6/12 (50.0%)
(95% CI)		(36.4–59.1)	(37.1–65.7)	(26.9–57.7)	(34.8–60.9)	(31.6–63.2)	(33.3–66.7)
**Unchanged**, n (%)		8/44 (18.2%)	5/35 (14.3%)	5/26 (19.2%)	5/23 (21.7%)	4/19 (21.1%)	0/12 (0.0%)
(95% CI)		(9.1–27.3)	(5.7–22.9)	(7.7–30.8)	(8.7–34.8)	(10.5–31.6)	–
**Worsened**, n (%)		1/44 (2.3%)	1/35 (2.9%)	0/26 (0.0%)	0/23 (0.0%)	1/19 (5.3%)	3/12 (25.0%)
(95% CI)		(0.0–6.8)	(0.0–8.6)	–	–	(0.0–15.8)	(8.3–41.7)
Total patients ^ѱ^	46/65 (70.8%)	44 (100%)	35 (100%)	26 (100%)	23 (100%)	19 (100%)	12 (100%)
Systolic BP (mmHg) Mean (± SD)	132.9 ± 15.0	125.4 ± 14.2	132.3 ± 15.6	132.2 ± 16.5	133.3 ± 17.2	131.4 ± 15.8	133.0 ± 19.4
Diastolic BP (mmHg) Mean (± SD)	76.0 ± 8.7	74.5 ± 8.6	72.7 ± 8.3	73.3 ± 8.8	75.4 ± 8.2	72.1 ± 9.5	72.5 ± 10.4
Antihypertensive therapy ^†^							
Yes, n (%)	44 (95.7%)	24 (58.5%)	19 (55.9%)	11 (50.0%)	12 (40.0%)	13 (72.2%)	9 (75.0%)
0	2 (4.3%)	15 (38.5%)	14 (42.4%)	10 (47.6%)	8 (40.0%)	5 (27.8%)	3 (25.0%)
1	13 (28.3%)	17 (43.6%)	12 (36.4%)	5 (23.8%)	6 (30.0%)	6 (33.3%)	3 (25.0%)
2	21 (45.7%)	4 (10.3%)	5 (15.2%)	5 (23.8%)	5 (25.0%)	6 (33.3%)	5 (41.7%)
3	4 (8.7%)	3 (7.7%)	1 (3.0%)	1 (4.8%)	1 (5.0%)	1 (5.6%)	1 (8.3%)
4	4 (8.7%)	0 (0.0%)	1 (3.0%)	0 (0.0%)	0 (0.0%)	0 (0.0%)	0 (0.0%)
5	2 (4.3%)	0 (0.0%)	0 (0.0%)	0 (0.0%)	0 (0.0%)	0 (0.0%)	0 (0.0%)
Mean number of drugs (± SD)	2.0 ± 1.1	0.9 ± 0.9	0.9 ± 1.0	0.9 ± 1.0	1.0 ± 0.9	1.2 ± 0.9	1.3 ± 1.0
Range	0–5	0–3	0–4	0–3	0–3	0–3	0–3

ѱ Patients with hypertension present at preoperative baseline. Bootstrap 95% confidence interval (CI) was computed by the bias-corrected and accelerated (BCa) method. † Beta blockers, angiotensin-converting enzyme (ACE) inhibitors, angiotensin receptor antagonists, calcium channel blockers and thiazide diuretics. *Abbreviations:* BP = blood pressure; SD = standard deviation. ^b^ No significant difference across the follow-up years with respect to changes in hypertension status (modelled from postoperative year 1 with generalised linear mixed model, *p* = 0.999).

#### 3.5.3. Hyperlipidaemia

Health improvements in hyperlipidaemia showed variability in response in contrast to T2DM and hypertension (Figure 3; Table 6). At preoperative baseline, 44 patients (67.7%) had hyperlipidaemia. Owing to the strict criteria of fulfilling discontinuation of lipid-lowering medications and normalization of the whole lipid profile comprising four subfractions (namely, total cholesterol, triglycerides, HDL-C and LDL-C), hyperlipidaemia remission and improvement rates over the postoperative years in the current study were relatively low.

Approximately half of the patients who continued with their current daily lipid-lowering agent treatment were considered to have persisting hyperlipidaemia (50.0–60.5%), as portrayed in Table 6. In contrast, there was an increasing linear trend of patients with worsening hyperlipidaemia from 2.6% to 30.0%. Notwithstanding, the number of patients remained at three individuals at years 5 and 6 post-surgery. As a result of the same strict criteria, hyperlipidaemia was in remission for 10.0~30.0% of study patients between year 1 and year 6 postoperatively. Similar proportions of patients experienced improvements across the follow-up timepoints as compared to remission (Table 6), which resulted in a composite cumulative remission and improvement rate of approximately 40.0% over long-term follow-up (Figure 3). Only one incidence requiring lipid-lowering therapy emerged de novo.

**Table 6:** Yearly remission, improved, persisting and worsened rates of hyperlipidaemia following bariatric surgery ^c^.

Follow-Up, Year	0	1	2	3	4	5	6
**Remission**, n (%)		6/38 (15.8%)	5/32 (15.6%)	6/24 (25.0%)	4/20 (20.0%)	5/17 (29.4%)	1/10 (10.0%)
(95% CI)		(7.9–23.7)	(6.3–25.0)	(12.5–37.5)	(10.0–30.0)	(17.6–47.1)	(0.0–30.0)
**Improved**, n (%)		8/38 (21.1%)	7/32 (21.9%)	4/24 (16.7%)	4/20 (20.0%)	3/17 (17.6%)	1/10 (10.0%)
(95% CI)		(10.5–31.6)	(12.5–31.3)	(4.2–29.2)	(10.0–30.0)	(5.9–29.4)	(0.0–30.0)
**Unchanged**, n (%)		23/38 (60.5%)	18/32 (56.3%)	12/24 (50.0%)	10/20 (50.0%)	6/17 (35.3%)	5/10 (50.0%)
(95% CI)		(47.4–73.7)	(40.6–71.9)	(37.5–66.7)	(30.0–70.0)	(17.6–52.9)	(30.0–70.0)
**Worsened**, n (%)		1/38 (2.6%)	2/32 (6.3%)	2/24 (8.3%)	2/20 (10.0%)	3/17 (17.6%)	3/10 (30.0%)
(95% CI)		(0.0–7.9)	(0.0–15.6)	(0.0–20.8)	(0.0–25.0)	(0.0–35.3)	(10.0–50.0)
Total patients ^ѱ^	44/65 (67.7%)	38 (100%)	32 (100%)	24 (100%)	20 (100%)	17 (100%)	10 (100%)
Total cholesterol, mmol/L	4.3 ± 1.2	4.8 ± 1.0	4.6 ± 1.3	4.7 ± 1.2	4.8 ± 1.0	4.7 ± 1.0	4.3 ± 1.3
Triglycerides, mmol/L	1.8 ± 0.9	1.5 ± 1.2	1.5 ± 1.2	1.3 ± 0.8	1.5 ± 0.9	1.7 ± 0.9	1.6 ± 0.9
HDL-C, mmol/L	1.1 ± 0.2	1.4 ± 0.3	1.4 ± 0.4	1.4 ± 0.4	1.4 ± 0.4	1.3 ± 0.3	1.3 ± 0.2
LDL-C, mmol/L	2.3 ± 1.1	2.7 ± 0.9	2.6 ± 1.1	2.7 ± 0.9	2.7 ± 0.9	2.5 ± 0.9	2.3 ± 1.0
Lipid-lowering drugs ^†^	N = 44	N = 41	N = 35	N = 23	N = 20	N = 19	N = 12
Yes, n (%)	35 (79.5%)	25 (61.0%)	22 (62.9%)	12 (52.2%)	11 (55.0%)	10 (52.6%)	9 (75.0%)
Mean number of drugs (± SD)	0.9 ± 0.6	0.7 ± 0.6	0.7 ± 0.6	0.6 ± 0.6	0.6 ± 0.6	0.7 ± 0.7	1.0 ± 0.7
Range	0–3	0–2	0–2	0–2	0–2	0–2	0–2

ѱ Patients with hyperlipidaemia present at preoperative baseline. Bootstrap 95% confidence interval (CI) was computed by the bias-corrected and accelerated (BCa) method. † Statin, fibrate and/or ezetimibe. *Abbreviations:* SD = standard deviation; HDL-C = high-density lipoprotein cholesterol; LDL-C = low-density lipoprotein cholesterol. ^c^ No significant difference across the follow-up years with respect to changes in hyperlipidaemia status (modelled from postoperative year 1 with generalised linear mixed model, *p* = 0.893).

Correspondingly, the lipid values and lipid-lowering drugs at all timepoints are reported (Table 6). With the control of the lipid profile, compared to baseline, amelioration was observed across all years after surgery—more so in triglyceride and HDL-C values but not in total cholesterol and LDL-C levels. The changes in the latter lipid levels were less marked, as if there was no improvement during follow-up. However, it is worth noting that these lipid parameters, specifically total cholesterol and LDL-C levels, reached normalization following our preoperative intensive lifestyle program and were sustained throughout postoperative follow-up. These levels likely plateaued in response to lipid-lowering agents thereafter, given that more than half of the patients continued therapy postoperatively. Despite being in the abnormal range at preoperative baseline, triglyceride markers achieved progressive improvement well after bariatric surgery throughout the 6-years follow-up period. Irrespective of the normal HDL-C level at preoperative baseline, the mean value increased progressively over follow-up timepoints. In this cohort, there was little cessation of lipid-lowering drugs, even in the setting of positive responses in terms of lipid parameters. These medications remain the recommended primary therapy for risk reduction in cardiovascular adverse events.

To further delineate the variability or equality of substantial metabolic changes in the whole sample across post-surgical follow-up, the estimated odds ratio in the four comorbidity statuses adjusted for sex, age at surgery and race were examined. The generalised linear mixed models accounting for random effects at the individual level revealed no significant difference (*p* > 0.05) in terms of modelled metabolic disease changes across the follow-up years (T2DM: *p* = 0.504; hypertension: *p* = 0.999; hyperlipidaemia: *p* = 0.893). This was following a significant remission and improvement in the three metabolic diseases 1 year after surgery (*p* < 0.001) (Figure 3; Table 4, Table 5 and Table 6). However, the comorbidity results, particularly year 6 post-surgery, should be interpreted with caveat in mind due to the small sample size and consequent uncertainty in the estimates.

#### 3.5.4. Sleep-Disordered Breathing

**Figure 4:**
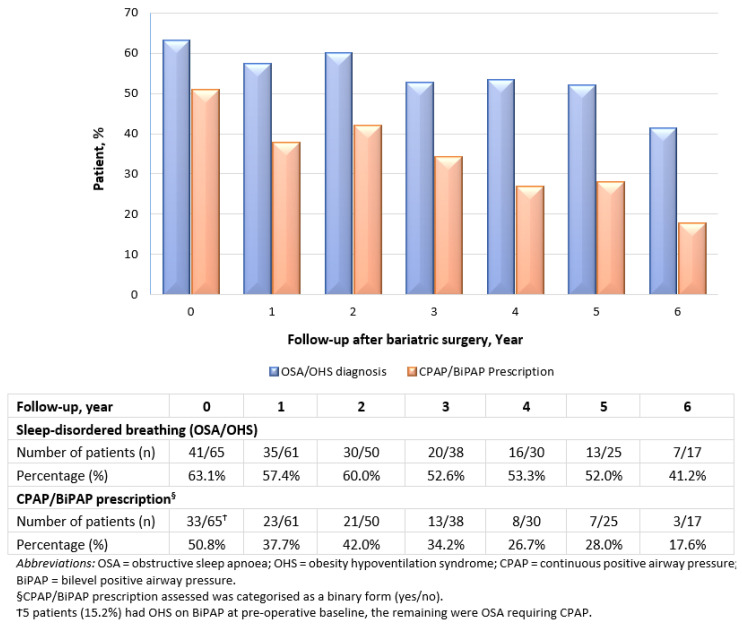
Prevalence of sleep-disordered breathing and CPAP/BiPAP prescription among the study population undergoing bariatric surgery.

The impact of bariatric surgery on sleep-disordered breathing and the use of CPAP/BiPAP devices is denoted in Figure 4. Prior to bariatric surgery, as many as 63.1% of patients who underwent bariatric surgery were diagnosed with either OSA or OHS. Ameliorations in OSA/OHS were observed to improve over timepoints, reducing to a prevalence of 41.2% at year 6 post-surgery. The prevalence of CPAP or BiPAP devices required by study patients was also observed to decrease tremendously from 50.8% at the time of bariatric surgery, with a slight upward trend at year 2, followed by a downtrend from year 3 onward and throughout the follow-up period to 17.6%. Aside from this, a total of 13 patients with OSA who were prescribed CPAP no longer required CPAP at their last observations following bariatric surgery, which is equivalent to a cumulative improvement rate of 39.4%. There was only one new onset of diagnosed mild OSA during the postoperative follow-up period. The CPAP/BiPAP adherence rate at preoperative baseline was 63.6%, i.e., 21 of 33 patients requiring CPAP/BiPAP. In other terms, 36.4% (12 patients) were not adherent to CPAP/BiPAP prescriptions before surgery. Patient self-reported factors found to influence non-progression to CPAP/BiPAP devices included intolerance to the machines, financial issues and breakdown of the machines.

#### 3.5.5. Opioid Use and Total Joint Arthroplasty

**Figure 5:**
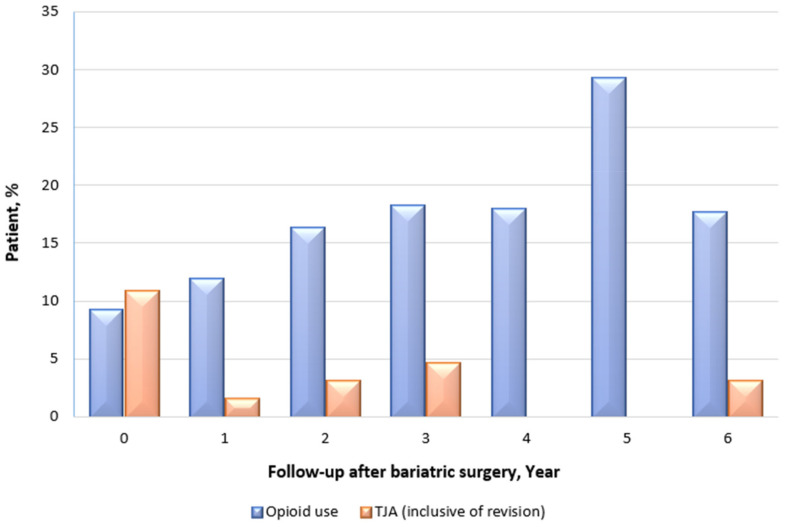

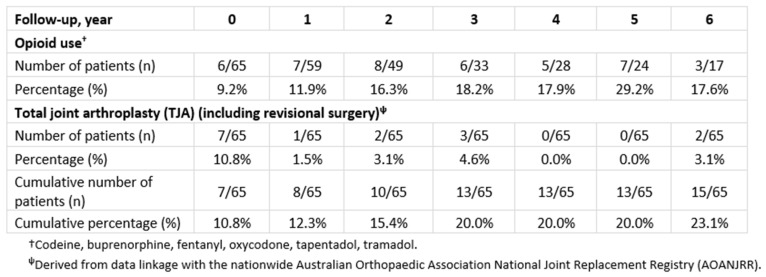
Prevalence of prescribed opioids and incidence of TJA among patients with OA-related joint pain.

Figure 5 shows the prevalence of prescribed opioid use and the cumulative incidence of TJA in the clinically severe obese patients with concurrent OA-related joint pain. At any given timepoint, the prevalence of opioid use across follow-ups was higher than before bariatric surgery (Figure 5). Opioid use or dependency increased annually post-surgery across all timepoints from baseline, particularly at postoperative year 5 (29.2%), despite a slight improvement in OA and localized pain. Specifically, patients with OA-related joint pain who were prescribed opioids at year 5 suffered from severe OA (n = 2), both OA and gout (n = 1), both OA and depression (n = 3), and both OA and degenerative disc disease (n = 1), which caused significant pain that required opioid pain relievers. Among those with OA-related joint pain (n = 49), 12.2% (n = 6) were prescribed opioids before bariatric surgery. Of the 6 patients who were prescribed opioids, 5 had ongoing opioid prescriptions through their last observations, whereas one persistent opioid user had discontinued medication used at the last observation. Therefore, patients with pre-diagnosed OA-related joint pain were predominantly prescribed non-opioid anti-inflammatory and pain-relief agents. Seven patients were post-surgery-initiated opioid users, i.e., post-surgery use of opioids without pre-surgery use. In contrast, the TJA rate decreased from 10.8% at baseline to 3.1% at year 6 post-bariatric surgery (i.e., 2 of 65 patients at year 6) according to data from the national joint replacement registry (i.e., AOANJRR). The registry also confirmed (not shown in Figure 5) that only one additional patient underwent TJA at year 7 and none in year 8 following bariatric surgery.

#### 3.5.6. Mental Illness and Use of Antidepressants and/or Antianxiety Agents

**Figure 6:**
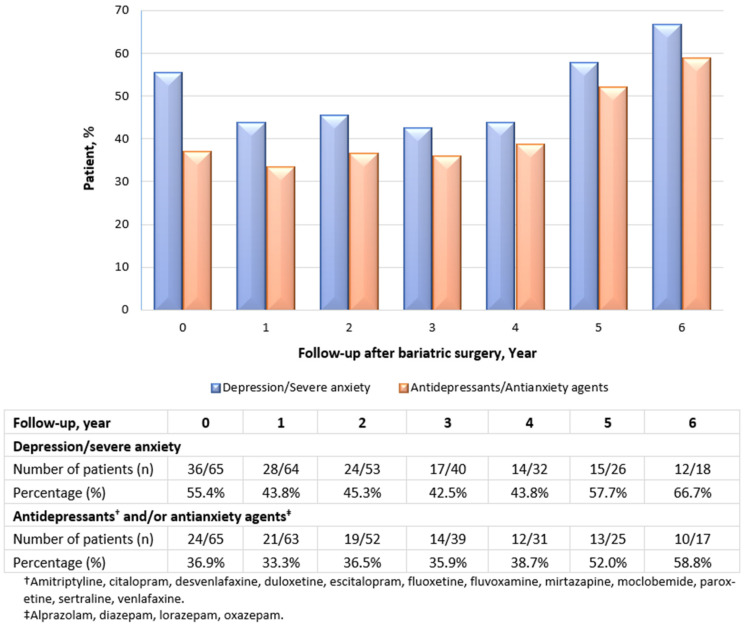
Prevalence of depression/severe anxiety, as well as the use of antidepressants and/or antianxiety agents among the study population.

Figure 6 shows the prevalence of mental illness, specifically depression/severe anxiety, as well as the use of antidepressants and/or antianxiety agents. More than half of patients (55.4%) were diagnosed with depression/severe anxiety prior to bariatric surgery. The prevalence dropped at years 1 to 4 post-surgery; it then increased and became slightly higher versus baseline by the fifth year (57.7%), reaching the highest rate at the sixth year postoperatively (66.7%) (Figure 6). Of the patients, 36.9% were on regular antidepressants and/or antianxiety agents prior to surgery. In agreement with the prevalence of depression and severe anxiety, the prevalence of use of the medications followed a quadratic trend—first, a decrease from baseline to years 1 to 3 post-surgery, followed by a gradual increase over time to a prevalence higher than baseline at years 4 and 5, reaching a new high at year 6.

### 3.6. Mortality

There was no death observed at any time in the 8 years of the post-surgical period.

## 4. Discussion

The burden of the epidemic of clinically severe obesity has prompted the development of a broad range of novel weight management options to support the efforts of those needing to lose weight and alleviate obesity-related comorbidities, with a consensus that bariatric surgery is at the top of the treatment pyramid. In this study, we present an in-depth longitudinal report of health outcomes from a publicly funded bariatric surgery service provided by two specialist obesity settings in NSW, Australia, with purpose-built physical clinical spaces and bariatric equipment. To date, no Australian report has presented such comprehensive and long-term data from public, multidisciplinary bariatric surgery settings, at least in NSW, in addition to linkage with the national orthopaedic joint registry. Our publicly funded bariatric surgery service adopting an intensive multidisciplinary approach has provided an ideal platform for research of the highest-grade obesity and its impacts in the severe and complex bariatric surgical population with at least three obesity-related comorbidities. We have addressed several knowledge deficiencies with respect to long-term weight change and the major obesity-related comorbidities following currently-performed bariatric surgeries. To achieve a finer understanding of the effects of bariatric surgery, clear changes in the status of metabolic diseases were computed based on laboratory findings, medications, physicians’ diagnoses and physical measurements to generate the prevalence, resolution, improvement, persistence, worsening and incidence rates.

Significant weight loss was achieved and sustained over the 9-year follow-up period from preoperative lifestyle intervention to post-surgery. Weight loss was maintained at 26.5 to 33.0 %TWL for 8 years following bariatric surgery, although there was a small degree of weight regain in the long term. This is in addition to another 13.2 %TWL during the preoperative preparation phase. Similar weight loss patterns were observed in the 5-year Finnish Sleeve vs. Bypass (SLEEVEPASS) randomized controlled trial (RCT) [10], the 3- and 7-year LABS studies [7,8], and the 6-year Utah Obesity study [28]. These patterns similarly involved an overall peak weight loss at 1 to 2 years after bariatric surgery, followed by a modest weight regain thereafter. This was despite ongoing annual clinic follow-up visits, and all our patients maintained > 20 %TWL postoperation to year 8. Several mechanisms of postoperative weight relapse have been proposed. These mechanisms are associated with mental health (e.g., depression, binge eating disorder, emotional eating, grazing and sweet cravings), maladaptive lifestyle behaviours (e.g., adherence to exercise and/or dietary recommendation), inadequate follow-up support, hormonal/metabolic imbalance (e.g., increased ghrelin levels) and anatomical/surgical factors (e.g., initial sleeve size, sleeve dilatation and amount of gastrointestinal track bypassed) [29,30,31].

Diabesity poses individual and global health challenges on an unprecedented scale. While bariatric surgery is recognised as a potent therapeutic option in people with diabesity, different gradients of efficiency have been reported in different studies [8,9,10,11,15,32,33,34,35,36,37,38]. In the current study, promising T2DM remission and improvement rates were observed. This was despite our cohort study being focused on a population with larger body size, multiple metabolic diseases, longstanding DM (mean duration of 10 years) and an average baseline HbA_1c_ ≥ 7.5%. The incidence of T2DM cases following bariatric procedures was zero at all follow-up assessment timepoints. The underlying mechanisms of T2DM remission and improvement after bariatric surgery involve the combined effects of incretin hormone secretion, bile acid metabolism, intestinal physiology, lipid regulation, neuronal signalling, microbiome changes and weight loss [39,40,41,42]. Our results are comparable to other longitudinal studies. This includes the Scandinavian Obesity Surgery Registry study [43], in which both glucose-lowering medication and glycaemic control endpoints were also observed. Although it was of shorter duration than our study, this study from Sweden reported that adults with diabesity who underwent bariatric surgery achieved 58.2% and 46.6% T2DM remission at 2 and 5 years after bariatric surgery, respectively [43]. Our findings are also similar to those reported in the United Kingdom National Bariatric Surgery Registry [44]. This study reported a 50% remission of T2DM over 5 years. However, no standardized clinical definitions or laboratory ranges were used to define remission. Instead, remission was dependent on the judgement of the clinicians submitting data to the registry; thus, the evidence was considered less convincing. Amongst landmark studies, the prospective matched SOS cohort study [15] revealed that surgically-treated patients had an excellent T2DM remission rate at 2 years post-bariatric surgery (72.3%, 219 of 303 patients); however the rate relapsed to 30.4% at 15 years post-surgery (35 of 115 patients). A similar outcome in terms of changes in T2DM statuses was reported in the prospective Swiss Multicentre Bypass or Sleeve Study (SM-BOSS) RCT (217 Swiss patients with clinically severe obesity, mean BMI = 43.9 kg/m^2^) [9], which reported proportions achieving T2DM remission (SG: 61.5% vs. RYGB: 67.9%), improvement (SG: 15.4% vs. RYGB: 7.1%), unchanged (SG: 11.5% vs. RYGB: 10.7%) and worsening (SG: 11.5% vs. RYGB: 14.3%) at 5 years postoperation. Our analyses of changes in T2DM statuses over 6 years of follow-up also corroborated the findings of a retrospective study of 74 Indiana patients who underwent LSG, which also reported a significant cumulative remission and improvement rate of 77% over the entire follow-up period [45]. The proportion achieving T2DM remission following bariatric surgery in the current study was in line with the results of the LABS study, which documented observed T2DM remission rates at 4, 5 and 7 years of 63.7%, 61.4% and 58.9%, respectively, for RYGB patients and 33.0%, 26.3% and 24.0%, respectively, for LAGB patients [8]. Despite the high severity of our patients’ obesity, the present findings demonstrate the significant ability of bariatric surgery to improve glucose homeostasis and induce T2DM remission. It should also be underlined that the study patients exhibited remarkably low worsening and incidence rates of T2DM despite the long follow-up period. Consequently, we support the already established position that bariatric surgery can serve as both the most effective treatment tool and an illuminating scientific model with which to address the diabesity crisis in the publicly funded healthcare system in the long term, even among highly severe patients with longstanding DM.

In this study, bariatric surgery also conferred substantial remission and improvement in hypertension. The observed decrease in medication use and blood pressure over the 6 years of follow-up reflects the substantial impact of bariatric surgery. These findings are consistent with numerous previous studies among patients who had undergone bariatric surgery. Our results are also in line with the Norwegian Prescription Database cohort study (median 6.5 years, range 0.2–10.1 years) among patients with clinically severe obesity, which reported that based on drug dispensation [35], remission of hypertension was observed in 31.9% of the surgically-treated group. Nevertheless, it is important to point out that using dispensed drugs as proxy outcomes, as was the case in this study, could result in underestimation of comorbidity rates; therefore, their results should be interpreted with caution. Regarding the high rate of cumulative remission and improvement of hypertension following bariatric surgery, similar results were observed in an Indiana study by Eid and colleagues. They reported a cumulative remission and improvement rate of 74% in hypertensive patients who underwent LSG [45]. This is in complete agreement with our findings that 74% of patients experienced major improvement or remission of hypertension over a 6-year follow-up period. As was found in the only studies with the full changes in statuses like our studies, the SM-BOSS RCT [9] showed that study patients exhibited higher rates of remitted, unchanged and worsened hypertension at 5 years postoperation than our study patients but not the improvement rate. The differences were likely due to the initial disease severity of the study cohorts, i.e., all of our study cohort had clinically complex obesity.

We also detailed progressive changes in hyperlipidaemia status by combining lipid-lowering medication use with all four crucial clinical serum lipid measures to address knowledge deficiencies with respect to the effectiveness of bariatric surgery on lipid profiles. In other terms, the definition of hyperlipidaemia remission used in the present study was four times stricter than studies that only assessed lipid subfractions separately [46,47]. This also resulted in the superiority of persistent hyperlipidaemia, i.e., among more than half of the study cohort, whereas one-third of the study patients attained either remission or improvement of hyperlipidaemia over the follow-up duration. Adipose tissue is defined as lipid storage, as well as an endocrinologically and immunologically active organ, and can be involved in the pathogenic mechanisms underlying hyperlipidaemia. Adipose tissue dysfunction is likely caused by obesity-induced stress [48], whereas bariatric surgery improves its function via a significant reduction in fat mass. In spite of this, the relationship between weight loss and lipid profile remains inconclusive. It has been proposed that improvement in the levels of HDL-C and triglycerides may be associated with weight loss [49], whereas LDL-C and total cholesterol might not. This theory exposes the mechanism of malabsorptive procedures that may tend to favour reductions in total cholesterol and LDL-C levels due to decreases in their absorption but not by SG or other restrictive procedures that were observed in the majority of our patients, which explains the plausibility of our findings with respect to increased HDL-C and lower triglycerides over the timepoints, but not the other lipid levels. Our data support previous studies that considered lipid variables but lacked data on lipid-lowering regimens and only reported short-term results [50]. The small change in measured total cholesterol and LDL-C levels observed in the present study was also likely plateaued by the use of lipid-lowering agents. Low rates of cessation of treatment in the current cohort over the study period, despite positive responses with respect to lipid parameters, were due to guideline recommendations for patients’ use in cardiovascular risk reduction, particularly in individuals with other cardiometabolic risk factors [51]. It is not possible to directly compare the results of the present study with those reported in the literature for several reasons. Apart from the different cut-offs and follow-up durations, the definitions used for hyperlipidaemia or dyslipidaemia vary widely. Some studies primarily evaluated medications alone [35,52,53]; others assessed some of the lipid parameters with no medication appraised at all [8,50]; some investigated lipid-lowering agents and lipid profiles separately without combining the outcomes as a comprehensive representation of hyperlipidaemia status [53]; some did not specify definitions at all or relied on patient self-reports [44,54]; and a couple of studies assessed medications but separated the lipid subfractions (e.g., hypercholesterolemia or hypertriglyceridemia) without evaluation of the whole lipid profile [46,47]. Only a few studies have reported hyperlipidaemia statuses, including remission, based on both lipid-lowering medications and lipid profiles; it is even rarer for all four fundamental lipid components to have been studied. The only longitudinal study that followed the same line as the present study is the 5-year SM-BOSS trial of patients with clinically severe obesity [9], with the exception that total cholesterol and lipid-lowering agents other than statins were not taken into consideration for the assessment of postoperative course. This difference potentially explains the contrasts between the findings of our population and this study, which reported higher remission and improvement of dyslipidaemia after SG (remission: 42.6%; improvement: 41.2%; unchanged: 16.2%; worsened: 0%) and RYGB (remission: 62.3%; improvement: 30.2%; unchanged: 7.5%; worsened: 0%). Therefore, other studies may not have identified more events of hyperlipidaemia remission and improvement if they had incorporated the strict definitions that we used.

Obesity is a modifiable risk factor for OA and weight-bearing joint pain [55]. Although relatively statistically constant, the clinical magnitude of the improvements in OA-related joint pain in our study patients, from 75.4% preoperatively to 68.4% following surgical weight loss over 6 years of follow-up, has provided important insights into the treatment. A 5-year observational study of 13 Pennsylvania patients with symptoms and radiographic evidence of knee OA who underwent bariatric surgery revealed that patients maintained improvements in knee symptoms, pain and daily living activities for 5 years following surgical weight loss, with only one patient undergoing knee replacement surgery (7.7%) [56]. The Western Ontario and McMaster Universities Index of Osteoarthritis and Knee Osteoarthritis Outcome Score Surveys were administered in this study. Despite the underpowered sample size of this study (n = 13), their results were similar to our findings at year 5 post-surgery, also highlighting maintenance of impressive improvements in OA and weight-bearing joint pain symptoms, as well as a very small number of patients undergoing TJA following bariatric surgery.

There is a dearth of research studying opioid use beyond the first year after bariatric surgery. The phenomenon observed in our study is in the same direction as that reported in the LABS-2 observational cohort study, which also revealed changes in the short- and long-term use of prescribed opioid analgesics following bariatric surgery. The LABS-2 study [57] (median BMI = 46 kg/m^2^) that also studied changes in prescribed analgesics post-surgery, reporting increased use of opioid medication following bariatric surgery, from 14.7% at preoperative baseline to 20.3%, i.e., above baseline levels, as time progressed at year 7. However, contrary to their proposed tandem that additional surgeries (such as back, knee, hip or ankle surgery, or a subsequent bariatric surgical procedure) were related to an increased risk of initiating opioid use following bariatric surgery, we found a reduced rate of TJA performed throughout the 6 follow-up years, including revisional surgeries, in the current investigation. Our study sheds further light on the beneficial effects of bariatric surgery on TJA other than symptomatic relief of OA-related joint pain, as observed in this cohort. In contrast to our medication data captured from hospital records and TJA data obtained from the national registry, the authors of the LABS-2 study noted their reliance on self-reported opioid analgesic medication use and that the accuracy in the sample was unmeasured. Despite this limitation, this study [57] and our findings are distinct from other studies that have reported relatively short time frames to examine the use of prescribed opioid medications after bariatric surgery [58,59,60,61]. We observed an increasing trend in the use of opioids in our study cohort post-surgery across nearly all timepoints, particularly year 5. The majority of the new opioid users did not suffer a surgical complication. Therefore, postoperative complications are unlikely to account for the development of new opioid use in our study cohort. Instead, our evaluation of indications for opioid prescriptions showed that opioid consumption was associated with patients’ significant pain caused by various deteriorated physiological and psychological outcomes. Understandably, these pains should not be disregarded nor undertreated. On the other hand, the bariatric surgical population may be more vulnerable to misuse of and dependence on opioids than the general population, given the risk of “addiction transfer” (i.e., exchange of one compulsive behaviour for another) [62], which may confer a similar misuse of opioids after surgery [63,64]. The full clinical significance of this aspect and its direct causal effect on this population was not explored in the current work and may warrant future studies. However, based on patients’ positive motivations for pursuing bariatric surgery and the risks associated with opioid abuse after surgery, we suggest healthcare providers should develop surveillance programs to monitor patients’ postoperative opioid use. This could be helpful in detecting patients at serious risk of misuse of opioids, facilitating prompt counselling of patients with respect to the risks, and identifying those at higher risk of poor surgical outcomes. Surgeons may also minimize patients’ exposure to such risks by following the current opioid prescribing guidelines while actively reducing excessive prescription of opioids. With much research demonstrating the benefits of weight loss with respect to OA, the problem still arises in daily surgical practice as to how best to combat this issue. Besides hospital policies preventing orthopaedic surgeons from performing TJA on some patients with severe obesity, the latter technically limits the ability to perform TJA [65]. We believe that more research in these areas should be conducted to determine optimal interventions for patients with both clinically severe obesity and OA symptoms, translating into practice to help those in need. To provide the most effective care possible, consideration should be given to referring patients to multidisciplinary services comprising orthopaedic surgeons, among other healthcare providers.

Our results suggest long-term decreases in the prevalence of OSA and OHS after bariatric surgery. The prevalence of use of CPAP or BiPAP devices required by the study patients decreased considerably from baseline to after surgery. Several other studies have also demonstrated the effectiveness of bariatric surgery in reducing OSA severity [66,67]. In a study of 132 clinically severe obese patients with OSA who underwent RYGB, the prevalence of OSA decreased from 71% at baseline to 44% at 1 year after bariatric surgery [67]. The authors also determined that OSA resolved in 45% of patients and improved in another 33% of the study patients [67]. These varying findings could potentially be explained by the differing definitions of OSA and its remission/improvement, different types of surgery, severities of patients or other differences in the nature of follow-up. Regardless of the controversy, bariatric surgery clearly demonstrates its additive effect on improvements in OSA and OHS.

It is noteworthy that our study cohort displayed a lower prevalence of depression/severe anxiety and the use of antidepressants and/or antianxiety agents in the first few years from pre-surgery but not years 5 and 6 post-surgery, at which the rate rebounded to a higher prevalence than the baseline. These findings verify the results reported in the LABS Consortium substudy [23], which reported a similar trend post-RYGB with respect to change in the prevalence of mental disorders over a 7-year follow-up period (n = 173). Significant short-term and the largest reductions in depression and anxiety within the first two years of bariatric surgery were also observed in the studies included in a recent systematic review (14 prospective studies of participants with BMI ≥ 35 kg/m^2^) [68]. Although this improvement may hold true, depression severity did increase after 2–3 years post-surgery, as shown in a meta-analysis of 58 studies [69]. Our longitudinal design provided confirmatory data that bariatric surgery offers short-term but not long-term improvements in depression and anxiety, supporting the proposed notion of a ‘psychological honeymoon‘ period of 3 years post-surgery, followed by rebounds in anxiety, depressive symptoms and binge eating disorder, as reported in other studies [22]. We recommend that all bariatric surgical candidates receive preoperative education and psychiatric evaluation as to the possibility of mental illness. This high-risk population should also be offered education on the symptoms of depression, as well as concrete steps to follow to get help and support if patients notice they are experiencing any depressive symptoms. Bariatric surgery is a life-saving and life-changing treatment. Unrealistic preoperative expectations about future weight reduction, environmental or social acceptability and obesity treatments may create a storm of serotonin depletion that leads to anxiety and depressive symptoms, anger, fatigue and irritability. These are some of the important areas that require further long-term investigation in the setting of clinically severe obesity.

### Limitations and Recommendations for Future Research

The study has some limitations that warrant discussion. First, the present study may be underpowered by its relatively small sample size related to the scarce resources of the public healthcare system and, therefore, stringent selection of the population, which may affect the ability to draw robust conclusions. Nonetheless, we were able to collect data from multiple resources and by several means with minimal missing data, as well as substantial in-depth variables in great detail. Additionally, we conducted modelling with repeated measures and inference tests up to 8 years of observations post-surgery, consisting of statistically adequate sample size and data. The results revealed meaningful patterns with respect to long-term changes in weight loss and obesity-related comorbidities in our study cohort. Secondly, only a minority of patients underwent OAGB and LAGB as index procedures in this study. Therefore, comparison between surgical procedures was not possible, and firm conclusions in this context could not be drawn. Even if we compared the three bariatric procedures in our study, the generalizability of the results might be compromised based on the fact that our study was dominated by patients who underwent SG. Thirdly, this study mainly included Caucasian patients with complex clinically severe obesity, which limits the generalizability of the results to populations of other races, those with less severe obesity or individuals not seeking specialist obesity treatments. Fourthly, the heterogeneity of outcome definitions in the field has also made combination and comparison across studies difficult; work toward standardization of outcome definitions would be beneficial.

## 5. Conclusions

This study confirms that bariatric surgery performed in public hospitals using an intensive multidisciplinary approach is effective and sustainable for long-term weight loss and the management of obesity-related comorbidities, even in highly severe and complex patients. This is one of the longest-term and most comprehensive reports encompassing a broad spectrum of aspects regarding bariatric surgery within multidisciplinary clinical obesity services across a wide range of up-to-date bariatric surgical procedures. The results of our study can serve as a foundation for shaping practice in other clinical settings, monitoring long-term patient outcomes, standardized clinical data recording, and future comparisons of multiple publicly funded bariatric surgery services. Major challenges concerning the successful outcomes of bariatric surgery in highly complex patients include maintaining mental health in the long run and reducing postoperative opioid use associated with patients’ significant pain caused by various deteriorated physiological and psychological outcomes. Improved access to bariatric surgery and long-term follow-up on a higher volume of patients are needed in the public healthcare system to better monitor patient outcomes, and drive positive changes in the overall delivery of multidisciplinary care.

## Figures and Tables

**Table 1:** Baseline sociodemographic and clinical characteristics of the study cohort by primary procedures.

Variable	Overall (n = 65)	SG (n = 52)	LAGB (n = 7)	OAGB (n = 6)
Age, mean ± SD (years)	54.2 ± 11.2	53.9 ± 11.7	57.3 ± 11.9	53.8 ± 5.6
Sex, n (%)				
Female	35 (53.8)	27 (51.9)	5 (71.4)	3 (50.0)
Male	30 (46.2)	25 (48.1)	2 (28.6)	3 (50.0)
Race, n (%)				
Caucasian	50 (76.9)	39 (75.0)	7 (100.0)	4 (66.7)
Middle Eastern	5 (7.7)	3 (5.8)	0 (0)	2 (33.3)
Other ^§^	10 (15.4)	10 (19.2)	0 (0)	0 (0)
Smoking status, n (%)				
Current/Ex-smoker	34 (52.3)	28 (53.8)	3 (42.9)	3 (50.0)
Never smoker	31 (47.7)	24 (46.2)	4 (57.1)	3 (50.0)
Excessive alcohol consumption ^¥^, n (%) (Current/Ex)	8 (12.3)	7 (13.5)	0 (0)	1 (16.7)
Employment status *, n (%)				
Employed	22 (33.8)	17 (32.7)	4 (57.1)	1 (16.7)
Unemployed ^#^	1 (1.5)	0 (0)	0 (0)	1 (16.7)
On government support payment ^†^, n (%)	37 (56.9)	30 (57.7)	3 (42.9)	4 (66.7)
Obesity categories, n (%)				
Class II (BMI < 40 kg/m^2^)	19 (29.2)	15 (28.8)	2 (28.6)	2 (33.3)
Class III (BMI ≥ 40 kg/m^2^)	46 (70.8)	37 (71.2)	5 (71.4)	4 (66.7)
Super obesity (BMI ≥ 50 kg/m^2^)	18 (27.7)	15 (28.8)	2 (28.6)	1 (16.7)

* *p* < 0.05 using chi-squared test or one-way ANOVA with post hoc Bonferroni test. *Abbreviations:* SG = sleeve gastrectomy; LAGB = laparoscopic adjustable gastric banding; OAGB = one anastomosis gastric bypass; SD = standard deviation; BMI = body mass index; kg = kilograms; m = metres. # Unemployment includes those who were not working or retired and not on any government support payment. † Disability support pension, age pension, carers pension, NewStart allowance, veteran pension, National Disability Insurance Scheme, workers compensation, Department of Housing or unemployment pension. § Indigenous Australian, Pacific Islander, Americas, Black African, Mauritian, Filipino and Pakistani. ¥ Excessive alcohol consumption is defined as ≥ 4 standard drinks most days of the week.

**Table 2:** Observed mean and median BMI change from initial clinic visit (lifestyle modifications and VLED) to pre-operation and follow-up year 8 after bariatric surgery.

	Initial	Preoperative Baseline	Postoperative Month 3	Postoperative Month 6	Postoperative Month 12	Postoperative Month 18	Postoperative Month 24	Postoperative Month 36	Postoperative Month 48	Postoperative Month 60	Postoperative Month 72	Postoperative Month 84	Postoperative Month 96
No. of patients	65	65	64	63	61	53	52	39	30	26	21	12	4
BMI (kg/m^2^)													
Mean (SD)	52.2 (12.5)	45.8 *** (8.8)	39.2 *** (7.6)	36.6 *** (6.9)	35.2 *** (6.9)	34.5 *** (7.1)	35.3 *** (7.3)	36.6 *** (7.5)	37.3 *** (7.2)	39.2 *** (7.5)	38.8 *** (7.0)	37.2 *** (6.3)	39.4 *** (8.3)
Median (IQR)	49.9 (11.7)	44.6 (11.1)	37.0 (10.2)	34.4 (9.0)	34.4 (9.0)	32.6 (8.3)	33.7 (10.0)	35.0 (8.8)	35.5 (9.9)	37.9 (11.2)	39.2 (12.7)	37.7 (11.4)	36.9 (14.4)
25th percentile	43.8	39.5	33.5	31.4	30.6	29.5	29.7	32.0	32.0	35.0	33.2	31.0	33.4
75th percentile	55.6	50.6	43.7	40.4	39.6	37.8	39.8	40.8	42.0	46.2	45.9	42.4	47.8

*** *p* < 0.001 versus initial baseline value. BMI is calculated as weight in kilograms divided by height in metres squared. *Abbreviations:* VLED = very low-energy diet; BMI = body mass index; kg = kilograms; m = metres; SD = standard deviation; IQR = interquartile range.

**Table 3:** Yearly clinical profiles of all study patients pre- and post-bariatric surgery (n = 65).

Follow-Up, Year	Baseline	1	2	3	4	5	6
**Clinical measurement**							
Systolic BP							
Mean (± SD) (mmHg)	129.5 ± 15.6	122.2 ± 14.5	127.6 ± 15.7	128.5 ± 17.4	132.4 ± 16.7	129.3 ± 16.2	130.9 ± 17.8
Diastolic BP							
Mean (± SD) (mmHg)	75.6 ± 8.9	73.5 ± 8.8	71.1 ± 9.1	74.0 ± 8.2	75.5 ± 8.1	72.8 ± 9.9	74.3 ± 10.1
**Laboratory parameters**							
HbA1_c_ (%)							
Mean (± SD)	7.2 ± 1.5	6.2 ± 1.1	6.3 ± 1.3	6.2 ± 1.2	6.7 ± 1.3	6.9 ± 1.3	7.0 ± 1.5
Range	(4.8–11.1)	(4.7–9.5)	(4.6–10.7)	(4.8–9.4)	(5.1–9.9)	(4.7–9.6)	(4.8–9.7)
FBG (mmol/L)							
Mean (± SD)	7.5 ± 2.6	6.2 ± 2.1	6.7 ± 2.3	6.2 ± 1.6	7.2 ± 2.1	7.2 ± 1.8	7.9 ± 2.9
Range	(4.3–18.4)	(4.3–14.8)	(4.3–14.7)	(4.1–11.5)	(4.6–11.5)	(4.7–10.7)	(4.9–15.9)
Total cholesterol, mmol/L	4.4 ± 1.1	4.7 ± 1.0	4.5 ± 1.2	4.7 ± 1.1	4.8 ± 1.0	4.6 ± 0.9	4.3 ± 1.2
Triglycerides, mmol/L	1.7 ± 0.8	1.4 ± 1.0	1.4 ± 1.0	1.3 ± 0.7	1.5 ± 0.9	1.7 ± 1.0	1.6 ± 0.8
HDL-C, mmol/L	1.2 ± 0.3	1.4 ± 0.3	1.4 ± 0.4	1.4 ± 0.3	1.4 ± 0.4	1.3 ± 0.2	1.3 ± 0.2
LDL-C, mmol/L	2.4 ± 1.0	2.7 ± 0.9	2.5 ± 1.0	2.7 ± 0.8	2.6 ± 0.9	2.4 ± 0.9	2.4 ± 0.9
**Use of medications**							
Antidiabetics	N = 65	N = 60	N = 50	N = 33	N = 28	N = 25	N = 17
Yes, n (%)	45 (69.2%)	21 (35.0%)	20 (40.0%)	10 (30.3%)	10 (35.7%)	13 (52.0%)	10 (58.8%)
Insulin treatment, n (%)	20 (30.8%)	7 (11.7%)	8 (16.0%)	5 (15.2%)	3 (10.7%)	2 (8.0%)	3 (17.6%)
Glucose-lowering agents, n (%)							
0	20 (30.8%)	42 (70.0%)	33 (66.0%)	24 (72.7%)	18 (64.3%)	12 (48.0%)	7 (41.2%)
1	23 (35.4%)	11 (18.3%)	10 (20.0%)	4 (12.1%)	5 (17.9%)	7 (28.0%)	3 (17.6%)
2	20 (30.8%)	6 (10.0%)	5 (10.0%)	3 (9.1%)	4 (14.3%)	4 (16.0%)	6 (35.3%)
3	2 (3.1%)	1 (1.7%)	2 (4.0%)	2 (6.1%)	1 (3.6%)	2 (8.0%)	1 (5.9%)
Mean number of drugs (± SD)	1.1 ± 0.9	0.4 ± 0.7	0.5 ± 0.8	0.5 ± 0.9	0.6 ± 0.9	0.8 ± 1.0	1.1 ± 1.0
Range	0–3	0–3	0–3	0–3	0–3	0–3	0–3
Antihypertensive therapy	N = 65	N = 59	N = 49	N = 33	N = 28	N = 24	N = 17
Yes, n (%)	44 (67.7%)	24 (40.7%)	19 (38.8%)	11 (33.3%)	12 (42.9%)	13 (54.2%)	9 (52.9%)
Mean number of drugs (± SD)	1.4 ± 1.3	0.6 ± 0.9	0.6 ± 0.9	0.6 ± 0.9	0.7 ± 0.9	0.9 ± 0.9	0.9 ± 1.0
Range	0–5	0–3	0–4	0–3	0–3	0–3	0–3
Lipid-lowering drugs, n (%)	N = 65	N = 60	N = 50	N = 34	N = 28	N = 24	N = 17
Yes, n (%)	35 (53.8%)	25 (41.7%)	22 (44.0%)	12 (35.3%)	11 (39.3%)	11 (45.8%)	10 (58.8%)
Mean number of drugs (± SD)	0.6 ± 0.7	0.5 ± 0.6	0.5 ± 0.6	0.4 ± 0.6	0.4 ± 0.6	0.6 ± 0.7	0.8 ± 0.8
Range	0–3	0–2	0–2	0–2	0–2	0–2	0–2

*Abbreviations:* BP = blood pressure; SD = standard deviation; HbA_1c_ = glycated haemoglobin; FBG = fasting blood glucose; HDL-C = high-density lipoprotein cholesterol; LDL-C = low-density lipoprotein cholesterol. N = total number of patients with data on the variables available at a given follow-up year.

**Table 4:** Yearly remission, improved, persisting and worsened rates of type 2 diabetes mellitus ^a^.

Follow-Up, Year	0	1	2	3	4	5	6
**Remission**, n (%)		26/50 (52.0%)	21/44 (47.7%)	18/33 (54.5%)	12/26 (46.2%)	7/22 (31.8%)	4/14 (28.6%)
(95% CI)		(40.0–64.0)	(34.1–61.4)	(39.4–69.7)	(30.8–61.5)	(18.2–50.0)	(14.3–42.9)
**Improved**, n (%)		15/50 (30.0%)	15/44 (34.1%)	9/33 (27.3%)	8/26 (30.8%)	8/22 (36.4%)	3/14 (21.4%)
(95% CI)		(20.0–40.0)	(22.7–45.5)	(15.2–39.4)	(15.4–46.2)	(22.7–50.0)	(7.1–35.7)
**Persisting**, n (%)		8/50 (16.0%)	7/44 (15.9%)	3/33 (9.1%)	4/26 (15.4%)	6/22 (27.3%)	4/14 (28.6%)
(95% CI)		(10.0–22.0)	(9.1–25.0)	(0.0–18.2)	(3.8–30.8)	(13.6–45.5)	(7.1–50.0)
**Worsened**, n (%)		1/50 (2.0%)	1/44 (2.3%)	3/33 (9.1%)	2/26 (7.7%)	1/22 (4.5%)	3/14 (21.4%)
(95% CI)		(0.0–6.0)	(0.0–6.8)	(0.0–18.2)	(0.0–19.2)	(0.0–13.6)	(0.0–42.9)
Total patients ^ѱ^	53/65 (81.5%)	50 (100%)	44 (100%)	33 (100%)	26 (100%)	22 (100%)	14 (100%)
HbA_1c_ (%)							
Mean (± SD)	7.5 ± 1.4	6.4 ± 1.1	6.5 ± 1.4	6.4 ± 1.3	6.9 ± 1.2	7.3 ± 1.0	7.3 ± 1.5
Range	(4.9–11.1)	(4.8–9.5)	(4.6–10.7)	(4.8–9.4)	(5.4–9.9)	(5.9–9.6)	(4.8–9.7)
FBG (mmol/L)							
Mean (± SD)	8.0 ± 2.6	6.6 ± 2.2	7.2 ± 2.4	6.6 ± 1.8	7.4 ± 2.1	7.5 ± 1.8	8.2 ± 3.1
Range	(4.9–18.4)	(4.3–14.8)	(4.5–14.7)	(4.1–11.5)	(4.8–11.5)	(5.1–10.7)	(4.9–15.9)
Antidiabetics							
Yes, n (%)	45 (84.9%)	21 (42.9%)	20 (50.0%)	10 (41.7%)	10 (47.6%)	13 (61.9%)	10 (76.9%)
Insulin treatment, n (%)	20 (37.7%)	7 (14.3%)	8 (20.0%)	5 (20.8%)	3 (14.3%)	2 (9.5%)	3 (23.1%)
Glucose-lowering agents ^†^, n (%)	N = 53	N = 49	N = 40	N = 24	N = 21	N = 21	N = 13
0	8 (15.1%)	31 (63.3%)	23 (57.5%)	15 (62.5%)	11 (52.4%)	8 (38.1%)	3 (23.1%)
1	23 (43.4%)	11 (22.4%)	10 (25.0%)	4 (16.7%)	5 (23.8%)	7 (33.3%)	3 (23.1%)
2	20 (37.7%)	6 (12.2%)	5 (12.5%)	3 (12.5%)	4 (19.0%)	4 (19.0%)	6 (46.2%)
3	2 (3.8%)	1 (2.0%)	2 (5.0%)	2 (8.3%)	1 (4.8%)	2 (9.5%)	1 (7.7%)
Mean number of drugs (± SD)	1.3 ± 0.8	0.5 ± 0.8	0.7 ± 0.9	0.7 ± 1.0	0.8 ± 1.0	1.0 ± 1.0	1.4 ± 1.0
Range	0–3	0–3	0–3	0–3	0–3	0–3	0–3

ѱ Patients with T2DM present at preoperative baseline. Bootstrap 95% confidence interval (CI) was computed by the bias-corrected and accelerated (BCa) method. † Biguanides, sulfonylureas, dipeptidyl peptidase 4 (DPP4) inhibitors, glucagon-like peptide-1 (GLP-1) receptor agonists, sodium-glucose transport protein 2 (SGLT2) inhibitors, α-glucosidase inhibitor and thiazolidinedione (TZD). *Abbreviations:* SD = standard deviation; HbA_1c_ = glycated haemoglobin; FBG = fasting blood glucose. ^a^ No significant difference across the follow-up years in terms of changes in T2DM status (modelled from postoperative year 1 with generalised linear mixed model, *p* = 0.504).

## Data Availability

The data are not publicly available due to the privacy of the patients involved and the restrictions imposed by the associated ethics approval. The data can be shared upon reasonable request to the corresponding authors.

## References

[1] Brightman L., Huang H.C.C., Dugdale P. (2019). Determining patient attendance, access to interventions and clinical outcomes in a publicly funded obesity programme: Results from the Canberra Obesity Management Service. Clin. Obes..

[2] Atlantis E., Kormas N., Samaras K., Fahey P., Sumithran P., Glastras S., Wittert G., Fusco K., Bishay R., Markovic T. (2018). Clinical obesity services in public hospitals in Australia: A position statement based on expert consensus. Clin. Obes..

[3] Aly A., Spiro C., Liu D.S., Mori K., Lim H.K., Blackham R., Erese R.J. (2022). Bariatric surgery in a public hospital: A 10-year experience. ANZ J. Surg..

[4] Backman B.B.D., Cottrell J., Campbell A., Clancy W., Halim Shah Y.J., Chadwick C., Budin A., MacCormick A., Caterson I., Brown W. (2020). The Bariatric Surgery Registry Annual Report, 2020.

[5] O’Brien P.E., MacDonald L., Anderson M., Brennan L., Brown W.A. (2013). Long-term outcomes after bariatric surgery: Fifteen-year follow-up of adjustable gastric banding and a systematic review of the bariatric surgical literature. Ann. Surg..

[6] Lainas P., Dammaro C., Gaillard M., Donatelli G., Tranchart H., Dagher I. (2018). Safety and short-term outcomes of laparoscopic sleeve gastrectomy for patients over 65 years old with severe obesity. Surg. Obes. Relat. Dis..

[7] Courcoulas A.P., Christian N.J., Belle S.H., Berk P.D., Flum D.R., Garcia L., Horlick M., Kalarchian M.A., King W.C., Mitchell J.E. (2013). Weight change and health outcomes at 3 years after bariatric surgery among individuals with severe obesity. JAMA.

[8] Courcoulas A.P., King W.C., Belle S.H., Berk P., Flum D.R., Garcia L., Gourash W., Horlick M., Mitchell J.E., Pomp A. (2018). Seven-year weight trajectories and health outcomes in the Longitudinal Assessment of Bariatric Surgery (LABS) study. JAMA Surg..

[9] Peterli R., Wölnerhanssen B.K., Peters T., Vetter D., Kröll D., Borbély Y., Schultes B., Beglinger C., Drewe J., Schiesser M. (2018). Effect of laparoscopic sleeve gastrectomy vs laparoscopic Roux-en-Y gastric bypass on weight loss in patients with morbid obesity: The SM-BOSS randomized clinical trial. JAMA.

[10] Salminen P., Helmiö M., Ovaska J., Juuti A., Leivonen M., Peromaa-Haavisto P., Hurme S., Soinio M., Nuutila P., Victorzon M. (2018). Effect of laparoscopic sleeve gastrectomy vs laparoscopic Roux-en-Y gastric bypass on weight loss at 5 years among patients with morbid obesity: The SLEEVEPASS randomized clinical trial. JAMA.

[11] Schauer P.R., Bhatt D.L., Kirwan J.P., Wolski K., Aminian A., Brethauer S.A., Navaneethan S.D., Singh R.P., Pothier C.E., Nissen S.E. (2017). Bariatric surgery versus intensive medical therapy for diabetes: 5-Year outcomes. N. Engl. J. Med..

[12] Akalestou E., Miras A.D., Rutter G.A., le Roux C.W. (2022). Mechanisms of weight loss after obesity surgery. Endocr. Rev..

[13] Mingrone G., Panunzi S., De Gaetano A., Guidone C., Iaconelli A., Capristo E., Chamseddine G., Bornstein S.R., Rubino F. (2021). Metabolic surgery versus conventional medical therapy in patients with type 2 diabetes: 10-Year follow-up of an open-label, single-centre, randomised controlled trial. Lancet.

[14] Sutanto A., Wungu C.D.K., Susilo H., Sutanto H. (2021). Reduction of Major Adverse Cardiovascular Events (MACE) after bariatric surgery in patients with obesity and cardiovascular diseases: A systematic review and meta-analysis. Nutrients.

[15] Sjöström L., Peltonen M., Jacobson P., Ahlin S., Andersson-Assarsson J., Anveden Å., Bouchard C., Carlsson B., Karason K., Lönroth H. (2014). Association of bariatric surgery with long-term remission of type 2 diabetes and with microvascular and macrovascular complications. JAMA.

[16] Sjöström L., Lindroos A.K., Peltonen M., Torgerson J., Bouchard C., Carlsson B., Dahlgren S., Larsson B., Narbro K., Sjöström C.D. (2004). Lifestyle, diabetes, and cardiovascular risk factors 10 years after bariatric surgery. N. Engl. J. Med..

[17] Plamper A., Lingohr P., Nadal J., Rheinwalt K.P. (2017). Comparison of mini-gastric bypass with sleeve gastrectomy in a mainly super-obese patient group: First results. Surg. Endosc..

[18] Ece I., Yilmaz H., Alptekin H., Yormaz S., Colak B., Yilmaz F., Sahin M. (2018). Comparative effectiveness of laparoscopic sleeve gastrectomy on morbidly obese, super-obese, and super-super obese patients for the treatment of morbid obesity. Obes. Surg..

[19] Alfadda A.A., Al-Naami M.Y., Masood A., Elawad R., Isnani A., Ahamed S.S., Alfadda N.A. (2021). Long-term weight outcomes after bariatric surgery: A single center Saudi Arabian cohort experience. J. Clin. Med..

[20] Singla V., Aggarwal S., Singh B., Tharun G., Katiyar V., Bhambri A. (2019). Outcomes in super obese patients undergoing one anastomosis gastric bypass or laparoscopic sleeve gastrectomy. Obes. Surg..

[21] Prachand V.N., Ward M., Alverdy J.C. (2010). Duodenal switch provides superior resolution of metabolic comorbidities independent of weight Loss in the super-obese (BMI ≥ 50 kg/m^2^) compared with gastric bypass. J. Gastrointest. Surg..

[22] Loh H.H., Francis B., Lim L.L., Lim Q.H., Yee A., Loh H.S. (2021). Improvement in mood symptoms after post-bariatric surgery among people with obesity: A systematic review and meta-analysis. Diabetes/Metab. Res. Rev..

[23] Kalarchian M.A., King W.C., Devlin M.J., Hinerman A., Marcus M.D., Yanovski S.Z., Mitchell J.E. (2019). Mental disorders and weight change in a prospective study of bariatric surgery patients: 7 Years of follow-up. Surg. Obes. Relat. Dis..

[24] Hawkins M., Leung S.E., Lee A., Wnuk S., Cassin S., Hawa R., Sockalingam S. (2020). Psychiatric medication use and weight outcomes one year after bariatric surgery. Psychosomatics.

[25] World Health Organization (WHO) (2019). Body Mass Index-BMI Geneva.

[26] American Diabetes Association (ADA) (2020). Standards of Medical Care in Diabetes—2020. Diabetes Care.

[27] Brethauer S.A., Kim J., El Chaar M., Papasavas P., Eisenberg D., Rogers A.M., Ballem N., Kligman M., Kothari S.N. (2015). Standardized outcomes reporting in metabolic and bariatric surgery. Obes. Surg..

[28] Adams T.D., Davidson L., Litwin S.E., Kolotkin R., LaMonte M.J., Pendleton R.C., Strong M.B., Vinik R., Wanner N.A., Hopkins P.N. (2012). Health benefits of gastric bypass surgery after 6 years. JAMA.

[29] Lauti M., Kularatna M., Hill A.G., MacCormick A.D. (2016). Weight regain following sleeve gastrectomy: A systematic review. Obes. Surg..

[30] Karmali S., Brar B., Shi X., Sharma A.M., de Gara C., Birch D.W. (2013). Weight recidivism post-bariatric surgery: A systematic review. Obes. Surg..

[31] Yu Y., Klem M.L., Kalarchian M.A., Ji M., Burke L.E. (2019). Predictors of weight regain after sleeve gastrectomy: An integrative review. Surg. Obes. Relat. Dis..

[32] Schauer P.R., Bhatt D.L., Kirwan J.P., Wolski K., Brethauer S.A., Navaneethan S.D., Aminian A., Pothier C.E., Kim E.S., Nissen S.E. (2014). Bariatric surgery versus intensive medical therapy for diabetes—3-year outcomes. N. Engl. J. Med..

[33] Courcoulas A.P., Belle S.H., Neiberg R.H., Pierson S.K., Eagleton J.K., Kalarchian M.A., DeLany J.P., Lang W., Jakicic J.M. (2015). Three-year outcomes of bariatric surgery vs lifestyle intervention for type 2 diabetes mellitus treatment: A randomized clinical trial. JAMA Surg..

[34] Hanipah Z.N., Schauer P.R. (2020). Bariatric surgery as a long-term treatment for type 2 diabetes/metabolic syndrome. Annu. Rev. Med..

[35] Jakobsen G.S., Småstuen M.C., Sandbu R., Nordstrand N., Hofsø D., Lindberg M., Hertel J.K., Hjelmesæth J. (2018). Association of bariatric surgery vs medical obesity treatment with long-term medical complications and obesity-related comorbidities. JAMA.

[36] Arterburn D.E., Bogart A., Sherwood N.E., Sidney S., Coleman K.J., Haneuse S., O’Connor P.J., Theis M.K., Campos G.M., McCulloch D. (2013). A multisite study of long-term remission and relapse of type 2 diabetes mellitus following gastric bypass. Obes. Surg..

[37] Madsen L.R., Baggesen L.M., Richelsen B., Thomsen R.W. (2019). Effect of Roux-en-Y gastric bypass surgery on diabetes remission and complications in individuals with type 2 diabetes: A Danish population-based matched cohort study. Diabetologia.

[38] Lager C.J., Esfandiari N.H., Luo Y., Subauste A.R., Kraftson A.T., Brown M.B., Varban O.A., Meral R., Cassidy R.B., Nay C.K. (2018). Metabolic parameters, weight loss, and comorbidities 4 years after Roux-en-Y gastric bypass and sleeve gastrectomy. Obes. Surg..

[39] Affinati A.H., Esfandiari N.H., Oral E.A., Kraftson A.T. (2019). Bariatric surgery in the treatment of type 2 diabetes. Curr. Diabetes Rep..

[40] Batterham R.L., Cummings D.E. (2016). Mechanisms of diabetes improvement following bariatric/metabolic surgery. Diabetes Care.

[41] Cho Y.M. (2014). A gut feeling to cure diabetes: Potential mechanisms of diabetes remission after bariatric surgery. Diabetes Metab. J..

[42] Penney N., Kinross J., Newton R., Purkayastha S. (2015). The role of bile acids in reducing the metabolic complications of obesity after bariatric surgery: A systematic review. Int. J. Obes..

[43] Jans A., Näslund I., Ottosson J., Szabo E., Näslund E., Stenberg E. (2019). Duration of type 2 diabetes and remission rates after bariatric surgery in Sweden 2007–2015: A registry-based cohort study. PLoS Med..

[44] Miras A.D., Kamocka A., Patel D., Dexter S., Finlay I., Hopkins J.C., Khan O., Reddy M., Sedman P., Small P. (2018). Obesity surgery makes patients healthier and more functional: Real world results from the United Kingdom National Bariatric Surgery Registry. Surg. Obes. Relat. Dis..

[45] Eid G.M., Brethauer S., Mattar S.G., Titchner R.L., Gourash W., Schauer P.R. (2012). Laparoscopic sleeve gastrectomy for super obese patients: Forty-eight percent excess weight loss after 6 to 8 years with 93% follow-up. Ann. Surg..

[46] Climent E., Benaiges D., Flores-Le Roux J.A., Ramon J.M., Pedro-Botet J., Goday A. (2018). Changes in the lipid profile 5 years after bariatric surgery: Laparoscopic Roux-en-Y gastric bypass versus laparoscopic sleeve gastrectomy. Surg. Obes. Relat. Dis..

[47] Vigilante A., Signorini F., Marani M., Paganini V., Viscido G., Navarro L., Obeide L., Moser F. (2018). Impact on dyslipidemia after laparoscopic sleeve gastrectomy. Obes. Surg..

[48] Bays H.E., Toth P.P., Kris-Etherton P.M., Abate N., Aronne L.J., Brown W.V., Gonzalez-Campoy J.M., Jones S.R., Kumar R., La Forge R. (2013). Obesity, adiposity, and dyslipidemia: A consensus statement from the National Lipid Association. J. Clin. Lipidol..

[49] Cunha F.M., Oliveira J., Preto J., Saavedra A., Costa M.M., Magalhães D., Lau E., Bettencourt-Silva R., Freitas P., Varela A. (2016). The effect of bariatric surgery type on lipid profile: An age, sex, body mass index and excess weight loss matched study. Obes. Surg..

[50] Spivak H., Sakran N., Dicker D., Rubin M., Raz I., Shohat T., Blumenfeld O. (2017). Different effects of bariatric surgical procedures on dyslipidemia: A registry-based analysis. Surg. Obes. Relat. Dis..

[51] Bibbins-Domingo K., Grossman D.C., Curry S.J., Davidson K.W., Epling J.W., García F.A., Gillman M.W., Kemper A.R., Krist A.H., Kurth A.E. (2016). Statin use for the primary prevention of cardiovascular disease in adults: US Preventive Services Task Force recommendation statement. JAMA.

[52] Pajecki D., Kawamoto F., Dantas A.C.B., Andrade P.C., Brasil N.C., Junqueira S.M., de Oliveira F.M.P., Ribeiro R.A., Santo M.A. (2020). Real-world evidence of health outcomes and medication use 24 months after bariatric surgery in the public healthcare system in Brazil: A retrospective, single-center study. Clinics.

[53] Patkar A., Fegelman E., RKashyap S., Brethauer S., Bour E., Yoo A., Li G. (2017). Assessing the real-world effect of laparoscopic bariatric surgery on the management of obesity-related comorbidities: A retrospective matched cohort study using a US Claims Database. Diabetes Obes. Metab..

[54] Wazir N., Arshad M.F., Finney J., Kirk K., Dewan S. (2019). Two years remission of type 2 diabetes mellitus after bariatric surgery. J. Coll. Physicians Surg. Pak..

[55] Gill R., Al-Adra D., Shi X., Sharma A., Birch D., Karmali S. (2011). The benefits of bariatric surgery in obese patients with hip and knee osteoarthritis: A systematic review. Obes. Rev..

[56] Hacken B., Rogers A., Chinchilli V., Silvis M., Mosher T., Black K. (2019). Improvement in knee osteoarthritis pain and function following bariatric surgery: 5-Year follow-up. Surg. Obes. Relat. Dis..

[57] King W.C., Chen J.-Y., Belle S.H., Courcoulas A.P., Dakin G.F., Flum D.R., Hinojosa M.W., Kalarchian M.A., Mitchell J.E., Pories W.J. (2017). Use of prescribed opioids before and after bariatric surgery: Prospective evidence from a US multicenter cohort study. Surg. Obes. Relat. Dis..

[58] Okifuji A., Hare B.D. (2015). The association between chronic pain and obesity. J. Pain Res..

[59] Raebel M.A., Newcomer S.R., Reifler L.M., Boudreau D., Elliott T., DeBar L., Ahmed A., Pawloski P., Fisher D., Donahoo W.T. (2013). Chronic use of opioid medications before and after bariatric surgery. JAMA.

[60] Raebel M.A., Newcomer S.R., Bayliss E.A., Boudreau D., DeBar L., Elliott T.E., Ahmed A.T., Pawloski P.A., Fisher D., Toh S. (2014). Chronic opioid use emerging after bariatric surgery. Pharmacoepidemiol. Drug Saf..

[61] Weingarten T.N., Sprung J., Flores A., Baena A.M.O., Schroeder D.R., Warner D.O. (2011). Opioid requirements after laparoscopic bariatric surgery. Obes. Surg..

[62] Brummett C.M., Waljee J.F., Goesling J., Moser S., Lin P., Englesbe M.J., Bohnert A.S.B., Kheterpal S., Nallamothu B.K. (2017). New persistent opioid use after minor and major surgical procedures in US adults. JAMA Surg..

[63] Bak M., Seibold-Simpson S.M., Darling R. (2016). The potential for cross-addiction in post-bariatric surgery patients: Considerations for primary care nurse practitioners. J. Am. Assoc. Nurse Pract..

[64] Smith M.E., Lee J.S., Bonham A., Varban O.A., Finks J.F., Carlin A.M., Ghaferi A.A. (2019). Effect of new persistent opioid use on physiologic and psychologic outcomes following bariatric surgery. Surg. Endosc..

[65] Hill D.S., Freudmann M., Sergeant J.C., Board T. (2018). Management of symptomatic knee osteoarthritis in obesity: A survey of orthopaedic surgeons’ opinions and practice. Eur. J. Orthop. Surg. Traumatol..

[66] Cohen J.B., Cohen D.L. (2015). Cardiovascular and renal effects of weight reduction in obesity and the metabolic syndrome. Curr. Hypertens. Rep..

[67] Peromaa-Haavisto P., Tuomilehto H., Kössi J., Virtanen J., Luostarinen M., Pihlajamäki J., Käkelä P., Victorzon M. (2017). Obstructive sleep apnea: The effect of bariatric surgery after 12 months. A prospective multicenter trial. Sleep Med..

[68] Gill H., Kang S., Lee Y., Rosenblat J.D., Brietzke E., Zuckerman H., McIntyre R.S. (2019). The long-term effect of bariatric surgery on depression and anxiety. J. Affect. Disord..

[69] Dawes A.J., Maggard-Gibbons M., Maher A.R., Booth M.J., Miake-Lye I., Beroes J.M., Shekelle P.G. (2016). Mental health conditions among patients seeking and undergoing bariatric surgery: A meta-analysis. JAMA.

